# Long-term health outcomes of Shiga toxin-producing *Escherichia coli* O157 (STEC O157) infection and STEC-associated haemolytic uraemic syndrome (STEC-HUS), Wales, 1990–2020

**DOI:** 10.1007/s00467-024-06640-x

**Published:** 2025-02-04

**Authors:** Rachel Merrick, Jiao Song, Laia Fina, Clare Sawyer, Claire Jenkins, Grace King, Drew Turner, Daniel Thomas, Christopher Williams

**Affiliations:** 1 Public Health Wales, Cardiff, UK; 2 UK Health Security Agency, London, UK

**Keywords:** STEC, STEC-HUS, *Escherichia coli*, Long-term, Chronic

## Abstract

**Background:**

Information on sequelae of Shiga toxin-producing *Escherichia coli* (STEC) O157 infection is limited to follow-up of paediatric haemolytic uraemic syndrome (HUS) cases. We investigate recorded long-term health outcomes experienced by individuals exposed to STEC O157 and STEC-HUS up to three decades on.

**Methods:**

We compared acute or new onset of chronic outcomes in individuals ≥ 1 year after STEC O157 or STEC-HUS to unexposed general population comparators between 01/01/1990–01/01/2019. The unexposed were their age- and sex-equivalents (4:1 matching ratio) and assigned the same study entry date. Outcomes were identified in primary and secondary care and categorised as kidney, neurological, cardiac, gastrointestinal, respiratory, or endocrine. Hazard ratios (HRs) and 95% confidence intervals (95% CI) were calculated using Cox regression.

**Results:**

Of 1,245 individuals with STEC O157, 65 developed HUS (5.2%). Individuals with STEC O157 were more likely to experience kidney (adjusted (a)HR: 1.9, 95% CI: 1.1–3.3), gastrointestinal (aHR: 1.7, 95% CI: 1.1–2.5) and respiratory (aHR: 1.4, 95% CI: 1.2–1.6) outcomes compared to the unexposed, on average between 3.4-11 years after exposure. Gastrointestinal (HR: 7.7, 95% CI: 2.6–23), kidney (HR: 5.5, 95% CI: 1.6–19), cardiac (HR: 5.1, 95% CI: 1.1–23) and respiratory (HR: 1.9, 95% CI: 1.1–3.1) outcomes were more common in the STEC-HUS cohort and occurred sooner, on average after 2.7-4.8 years.

**Conclusions:**

Long-term complications were nearly twice as likely in the STEC O157 cohort, and as many as eight times more likely following STEC-HUS. We recommend that those exposed to STEC be monitored for at least five years for late-emerging kidney and extrarenal complications.

**Graphical abstract:**

**Figure:**
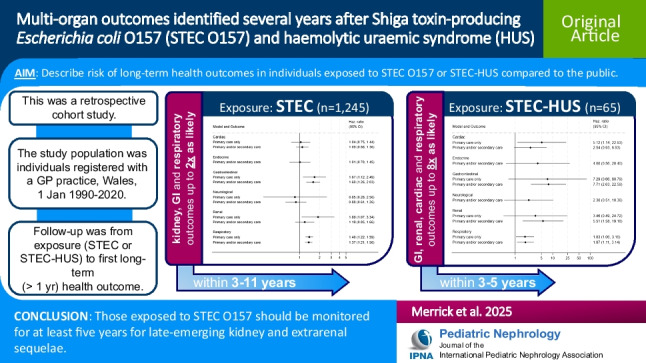
A higher resolution version of the Graphical abstract is available as Supplementary information

**Supplementary Information:**

The online version contains supplementary material available at 10.1007/s00467-024-06640-x.

## Introduction

### Shiga toxin-producing *Escherichia coli*

Shiga toxin-producing *Escherichia coli* (STEC) is a potentially serious zoonotic cause of gastrointestinal disease in humans [1]. Transmission from animals to humans is primarily foodborne, including from undercooked ground beef, raw produce, and unpasteurised milk [2]. The predominant serotype is O157:H7, which is estimated to be responsible for more than one million cases of diarrhoea worldwide annually [3]. In England and Wales, an average of 887 cases of STEC O157 were reported annually between 1997–2012, decreasing to 539 cases in 2019 [4, 5].

STEC O157 is of major public health concern as it can cause severe symptoms such as bloody diarrhoea and haemolytic uraemic syndrome (HUS), a leading cause of kidney failure in children and cause of acute kidney injury in adults [6]. While rare, fatalities can occur, as was observed in a devastating foodborne outbreak across 44 schools in South Wales in 2005 [7]. There is growing awareness that non-O157 serogroups can also cause severe disease [8, 9]. However, historical data on these serogroups are lacking because the detection of STEC non-O157 has only been made possible relatively recently following diagnostic developments [5].

### Rationale for studying complications of STEC O157

Although there is a large body of literature on STEC O157, information on sequelae is limited to follow-up of paediatric HUS cases with complications involving the kidneys and central nervous system [10–12]. Less commonly studied are infections which do not progress to HUS, as well as extrarenal complications beyond the acute phase. However, as Shiga toxins produced by *Escherichia coli* may damage the colon and contribute to a wide range of complications irrespective of HUS [13], a need for long-term follow-up after STEC infection to assess potentially late-emerging sequelae has been identified [6, 10, 14, 15]. In this study, we describe the long-term health complications experienced by individuals exposed to STEC O157, associated with and without HUS, and compare them to members of the public to inform clinical management and burden of disease estimates. We focus on STEC O157, as opposed to non-O157, due to the availability of longitudinal data for the former.

## Methods

### Study design

We conducted a retrospective matched cohort study [16, 17], comparing recorded health outcomes for individuals ≥ 1 year after STEC O157 or STEC-HUS to their unexposed equivalents in the general population.

### Study setting and period

All individuals resident in Wales and registered with a General Practitioner (GP) practice between 1 January 1990 and 1 January 2020.

### Exposed population

Exposed persons were defined upon:

1. Laboratory confirmation of STEC O157 infection with the earliest of positive specimen or symptom onset date between 1 January 1990 and 1 January 2019
2. The above, with an International Classification of Diseases (ICD-10) coded hospital episode of D59.3 (HUS) within 28 days of the earliest of STEC O157 positive specimen or symptom onset date

A conservative 28-day criterion was included in the exposure definition for STEC-HUS as HUS has been described to occur in 5–10% of individuals with STEC within one week of the onset of gastrointestinal symptoms [18].

### Unexposed population

All individuals in the general population who did not have laboratory confirmation of STEC O157 infection.

### Selection of the unexposed sample

A sampling frame of eligible matches on week of birth and sex was created from the unexposed population for each exposed person. Eligible matches were assigned and sorted on a randomly generated number. The first four matches in each sampling frame were kept. Each matched unexposed person was assigned the same date of study entry as their exposed counterpart, which was the earliest of positive specimen or symptom onset date of STEC O157 infection.

### Exclusion criteria

Those exposed and their eligible matches in the unexposed sample were excluded if they were reported to have died or deregistered from a GP practice in Wales within the first year of study entry. Individuals were excluded if their infection was known to be non-O157 STEC.

### Endpoint

The first of any of the following ≥ 1 year after study entry: an acute or new onset of a chronic condition (hereby referred to as a health outcome), deregistration from a GP practice in Wales (hereafter referred to as emigrated), death, or study end (1 January 2020). Long-term was defined as endpoints achieved ≥ 1 year after study entry, as has been used previously [11]. New onset was defined as first report of a chronic condition ≥ 1 year after study entry.

### Health outcomes

Health outcomes were selected based on existing literature, most notably a review by Spinale and colleagues [6], and the expert opinion of consultants in microbiology and infectious diseases. Outcomes were broadly categorised for ease of reporting as kidney (chronic kidney disease, kidney failure), neurological (epilepsy, cognitive impairment), cardiac (hypertension, ischaemic heart disease), gastrointestinal (irritable bowel syndrome, inflammatory bowel disease), respiratory (acute respiratory tract infections), or endocrine (diabetes).

Outcomes were stratified on data source as 1) primary care only and 2) primary and/or secondary care. This was because the former was hypothesised to be a proxy measure of milder disease. In Wales, most patients initially consult their GP practice (i.e., primary care) for non-emergency medical issues. Patients may then be referred for specialist care (i.e., secondary care) depending on the complexity and urgency of the condition being managed. Associated Read v2 and ICD-10 clinical code lists were obtained from previous publications and are listed in full under the relevant category in Supplementary Appendix A, B [19–27]. Individuals newly diagnosed with diabetes (type 1, type 2, undetermined) were identified using a validated algorithm [28].

### Data sources

A linelist of individuals with laboratory confirmed STEC O157 was compiled from historic national surveillance datasets held by Public Health Wales and the Gastrointestinal Bacteria Reference Unit at the UK Health Security Agency. These data were anonymised by NHS Wales Informatics Service and imported into a privacy-protecting trusted research environment, the Secure Anonymised Information Linkage (SAIL) Databank, using a split-file approach described elsewhere [29, 30]. The SAIL Databank is hosted by Swansea University and contains anonymised individual-level, population scale data. We linked the anonymised linelist of STEC O157 cases to the following in SAIL:

- Annual District Death Extract; for date of death
- Critical Care Dataset; for date of critical care attendance
- Emergency Department Dataset; for date of emergency department attendance
- Outpatient Referrals from Primary Care; for date of outpatient attendance
- Patient Episode Database for Wales; for date of hospital admission and associated ICD-10 coded episodes
- Welsh Demographic Service Dataset; for week of birth, sex, GP practice registration dates, and Welsh Index of Multiple Deprivation (WIMD) quintile version 2019
- Welsh Longitudinal General Practice Dataset; for date of GP practice attendance and associated Read v2 coded consultations

The Welsh Demographic Service Dataset was also used to identify the unexposed sample. WIMD quintile version 2019 is a measure of multiple deprivation and is based on residential address in Wales (1 = most deprived, 5 = least deprived).

### Matching criteria and co-variates

Matching criteria (age, sex) and covariates (WIMD and co-morbidity) were assessed at time of study entry. Charlson co-morbidity scores were assigned to individuals based on their historical ICD-10 codes from secondary care attendances [31]. A list of co-morbidities considered is available in Supplementary Appendix C. Attendance at secondary care during the acute episode was also assessed at study entry but was not included as a covariate due to collinearity with STEC-HUS. Healthcare-seeking behaviour was based on presentation at any of critical care; an emergency department; an outpatient department; or hospital admission within 28 days of study entry date.

### Data management

SAIL datasets were linked on a unique, anonymous identifier, which itself was obtained using an iterative matching process described fully elsewhere [30]. Data were imported into Stata version 18.0 [32] and RStudio version 4.1.3 [33] for cleaning, analysis, and visualisation. Age was reported as a median value for the STEC-HUS cohort due to small sample size. Charlson co-morbidity score was categorised as zero/one or more for the STEC-HUS cohort for the same reason. Values less than five and associated percentages or incidence rates were masked, aggregated or omitted where deductive disclosure was possible. Follow-up time was presented as median values to prevent back-calculation of the number with each health outcome. The results of the model of neurological outcomes in the STEC-HUS cohort, as recorded in primary care only, were not reported due to small sample size. As endocrine outcomes were determined using an algorithm which used primary and secondary care data, we did not run the primary care only model for endocrine outcomes.

### Data analysis

#### Descriptive

The demographic and clinical characteristics of the exposed populations and unexposed samples were described and compared for statistical differences (*p* ≤ 0.05) using the t-test or a non-parametric test equivalent for continuous data. Fisher’s exact or Chi squared test was used for categorical data. Continuous data were assessed for normality through histograms and the Shapiro–Wilk test. Data were summarised with the mean and standard deviation or median and inter-quartile range (IQR). Categorical variables were presented in frequency tables with percentages. Missing data were described in tabular form.

Individuals were censored if they emigrated, died, or did not experience a health outcome prior to study end (1 January 2020). Follow-up time was not resumed if an individual re-registered with a GP practice in Wales > 30 days after their deregistration date as it was not possible to ascertain if an endpoint was achieved during the lost to follow-up period. Incidence rates (IR) were calculated per 10,000 person-years at risk. Incidence rate ratios (IRR) and associated *p*-values were calculated as: IR (exposed)/IR (unexposed). Person-years at risk prior to censoring was included in analyses.

#### Univariate

Hazard ratios (HRs) and 95% CI were calculated to investigate the effect of each exposure (STEC O157 and STEC-HUS) on time-to-event using Cox proportional hazards regression for matched cohort data [16, 17]. Models were stratified by health outcome (kidney, neurological, cardiac, gastrointestinal, respiratory, endocrine) and data source (primary care only, primary and/or secondary care). The proportional hazards assumption was checked using statistical tests and graphical diagnostics based on the scaled Schoenfeld residuals. *P*-values were obtained using the Wald test.

#### Multivariable

Multivariable models were created for the STEC O157 cohort using the approach described above, with additional inclusion of covariates. Covariates were removed from each model using a backwards stepwise approach. Covariates were excluded from the final models if the Akaike information criterion value decreased with their removal.

### Ethical approval

Research ethics approval was granted by Health Research Authority (HRA)/Health and Care Research Wales (HCRW) in February 2019 (IRAS Project ID: 243461). Minor changes to the study were reapproved by HRA/HCRW in January 2023. The study was approved by the SAIL Information Governance Review Panel (Project 1531). All data containing patient identifiable information were handled and stored in compliance with the Data Protection Act (2003) and GDPR (2018).

## Results

### STEC O157

#### Exposed

**Fig. 1:**
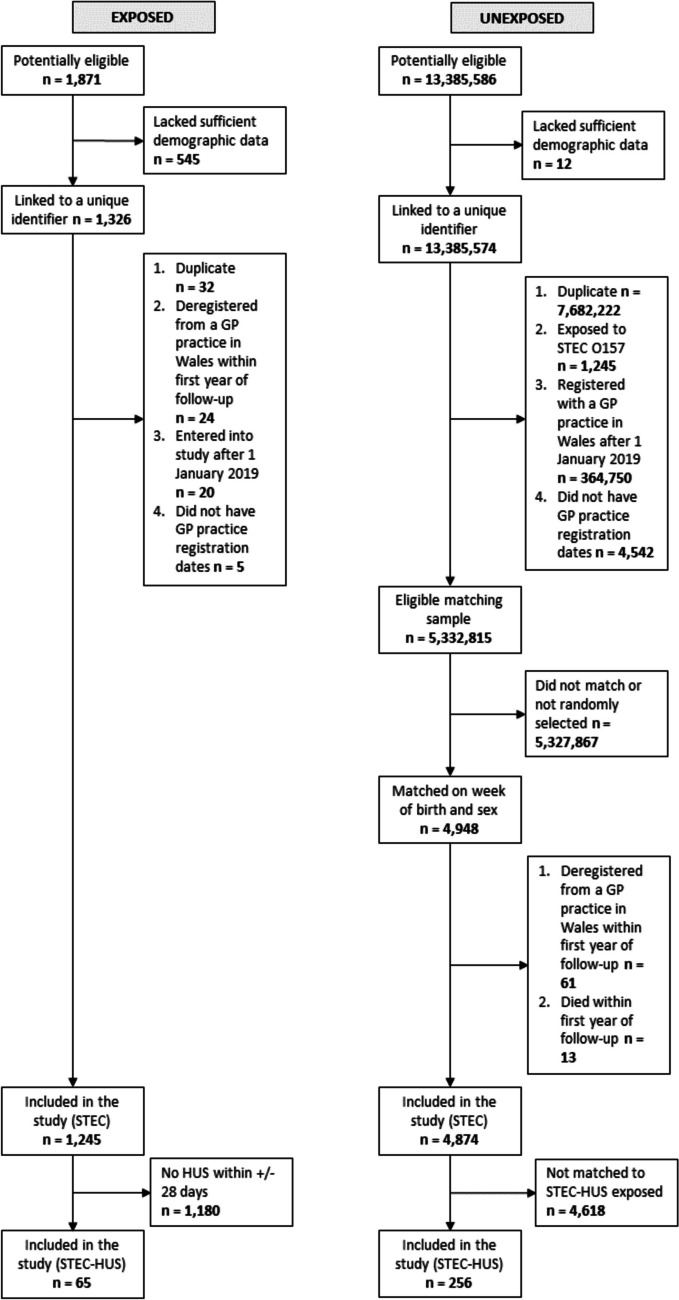
Flow diagram of the number of individuals at each stage of the study. Exposure was to Shiga toxin-producing *Escherichia coli* O157 infection (STEC O157) or STEC O157 with haemolytic uraemic syndrome (HUS), Wales, 1990–2020. The unexposed cohort comprised individuals registered with a General Practitioner practice in Wales who were the same age and sex as their exposed equivalent at time of STEC O157 positive specimen date or symptom onset

Among 1,871 potentially eligible individuals, 1,326 had sufficient demographic data to be linked to a unique identifier, of which 1,245 were included in the STEC O157 analysis (Fig. 1). The median age was 19 years (IQR: 4–42 years) (Table 1). The majority were female (682/1,245, 55%). One third (402/1,245, 32%) sought hospital care in the acute episode. At ≥ 1 year after study entry, there were 79 (6.3%) who were reported to have died and 103 (8.3%) who had emigrated (Table 1).

**Table 1:** Characteristics of study participants. Exposure was to Shiga toxin-producing *Escherichia coli* O157 infection (STEC O157), Wales, 1990–2020. The unexposed cohort comprised individuals registered with a General Practitioner practice in Wales who were the same age and sex as their exposed equivalent at time of STEC O157 specimen or symptom onset date

	Study group		
	Unexposed, *N* = 4,874^1^	Exposed, *N* = 1,245^1^	*p*-value^2^
**Study entry**			> 0.9
January-March	463 (9.5%)	118 (9.5%)	
April-June	1,124 (23%)	286 (23%)	
July–September	2,309 (47%)	591 (47%)	
October-December	978 (20%)	250 (20%)	
**Age (years)**	19 (4, 42)	19 (4, 42)	0.9
**Age group (years)**			> 0.9
≤ 5	1,492 (31%)	380 (31%)	
6–10	531 (11%)	134 (11%)	
11–15	236 (4.8%)	59 (4.7%)	
16–19	213 (4.4%)	56 (4.5%)	
20–29	571 (12%)	148 (12%)	
30–39	505 (10%)	128 (10%)	
40–49	334 (6.9%)	84 (6.7%)	
50–59	405 (8.3%)	102 (8.2%)	
≥ 60	587 (12%)	154 (12%)	
**Gender**			0.8
Male	2,208 (45%)	558 (45%)	
Female	2,666 (55%)	682 (55%)	
**Index of multiple deprivation**			< 0.001
Q5 (least deprived)	907 (19%)	241 (19%)	
Q4	942 (19%)	270 (22%)	
Q3	995 (20%)	284 (23%)	
Q2	924 (19%)	254 (20%)	
Q1 (most deprived)	1,106 (23%)	196 (16%)	
**Co-morbidity score**			< 0.001
Zero	4,162 (95%)	1,129 (91%)	
One	169 (3.5%)	73 (5.9%)	
Two or more	93 (1.9%)	43 (3.5%)	
**Haemolytic uraemic syndrome**	0 (0%)	65 (5.2%)	< 0.001
**Sought care in acute episode**	120 (2.5%)	402 (32%)	< 0.001
**Died**	285 (5.8%)	79 (6.3%)	0.5
**Emigrated**	796 (16%)	103 (8.3%)	< 0.001
**Kidney**			
Primary care only			
Outcome	63 (1.3%)	21 (1.7%)	0.3
Time to outcome (years)	8.3 (4.8, 13.9)	7.5 (2.6, 11.6)	0.4
Primary and/or secondary care			
Outcome	185 (3.8%)	54 (4.3%)	0.4
Time to outcome (years)	8.2 (4.5, 13.6)	8.8 (2.9, 15.2)	> 0.9
**Neurological**			
Primary care only			
Outcome	21 (0.4%)	5 (0.4%)	0.9
Time to outcome (years)	7.5 (5.1, 16.4)	6.2 (4.9, 10.0)	0.6
Primary and/or secondary care			
Outcome	171 (3.5%)	41 (3.3%)	0.7
Time to outcome (years)	8.0 (4.7, 16.6)	8.9 (4.9, 12.6)	0.7
**Cardiac**			
Primary care only			
Outcome	170 (3.5%)	50 (4.0%)	0.4
Time to outcome (years)	8.2 (4.3, 13.5)	9.5 (4.6, 13.6)	0.6
Primary and/or secondary care			
Outcome	460 (9.4%)	127 (10%)	0.4
Time to outcome (years)	7.0 (3.6, 12.2)	7.6 (2.9, 13.6)	0.9
**Gastrointestinal**			
Primary care only			
Outcome	91 (1.9%)	39 (3.1%)	0.006
Time to outcome (years)	8.1 (3.6, 13.3)	10.6 (5.7, 12.5)	0.3
Primary and/or secondary care			
Outcome	278 (5.7%)	106 (8.5%)	< 0.001
Time to outcome (years)	7.5 (4.0, 12.6)	7.7 (3.3, 11.9)	0.4
**Respiratory**			
Primary care only			
Outcome	1,016 (21%)	340 (27%)	< 0.001
Time to outcome (years)	5.3 (2.6, 10.4)	4.3 (2.2, 8.6)	0.004
Primary and/or secondary care			
Outcome	1,122 (23%)	371 (30%)	< 0.001
Time to outcome (years)	4.3 (1.9, 8.3)	3.4 (1.8, 6.8)	0.4
**Endocrine**			
Primary and/or secondary care			
Outcome	177 (3.6%)	41 (3.3%)	0.6
Time to outcome (years)	7.4 (4.4, 12.2)	9.2 (4.5, 13.7)	0.13

^1^*n* (%); Median(IQR)

^2^Pearson’s Chi-squared test; Wilcoxon rank sum test; Wilcoxon rank sum exact test

The median follow-up time is reported for each model in Table 2, ranging from 9.2–12 years. Considering the first outcome recorded in primary and/or secondary care ≥ 1 year after study entry, the incidence of respiratory outcomes was the highest (288 per 10,000 person-years at risk), followed by cardiac (86 per 10,000 person-years at risk), gastrointestinal (69 per 10,000 person-years at risk), kidney (34 per 10,000 person-years at risk), endocrine (26 per 10,000 person-years at risk) and neurological (26 per 10,000 person-years at risk) outcomes. The most common, first recorded outcome in each category of disease was acute respiratory tract infection (288 per 10,000 person-years at risk), hypertension (64 per 10,000 person-years at risk), irritable bowel syndrome (45 per 10,000 person-years at risk), chronic kidney disease (stages 3–5) (12 per 10,000 person-years at risk), type 2 diabetes (20 per 10,000 person-years at risk) and cognitive impairment (21 per 10,000 person-years at risk), respectively. Outcomes only recorded in primary care followed the same trend in terms of most and least common, but the incidence rates were lower. Disease-specific incidence rates are available in Supplementary Appendix D.

**Table 2:** Counts, median person-time at risk (years), and incidence rates for outcomes (kidney, neurological, cardiac, gastrointestinal, respiratory, endocrine) among individuals exposed to Shiga-toxin producing *Escherichia coli* O157 infection (STEC O157) and STEC O157 associated with haemolytic uraemic syndrome (STEC-HUS), Wales, 1990–2020. Outcomes were defined using clinical code lists for General Practitioner practice consultations (“Primary care only”) and/or hospital admissions (“Primary and/or secondary care”). Unexposed cohorts were individuals registered with a General Practitioner practice in Wales who were the same age and sex as their exposed equivalent at time of STEC O157 specimen or symptom onset date

Model	Exposure	Outcome	Exposed				Unexposed				Overall	
			*N*	(%)	Time at risk (person-years)^1^	Incidence (per 10,000 person-years)	*N*	(%)	Time at risk (person-years)^**1**^	Incidence (per 10,000 person-years)	Incidence rate ratio	*p*-value
**Kidney**												
	STEC	Primary care only	21	1.7	12.2	13.1	63	1.3	11.6	10.4	1.3	0.356
		Primary and/or secondary care	54	4.3	11.8	34.1	185	3.8	11.5	30.7	1.1	0.492
	STEC-HUS	Primary care only	< 5	< 7.7	11.3	28.0	< 5	< 2.0	11.3	10.6	2.7	0.323
		Primary and/or secondary care	< 10	< 15.4	10.8	103.0	< 10	< 3.9	11.3	21.1	4.9	0.007
**Neurological**												
	STEC	Primary care only	5	0.4	12.4	3.1	21	0.4	11.7	3.4	0.9	0.869
		Primary and/or secondary care	41	3.3	12.1	25.7	171	3.5	11.5	28.4	0.9	0.585
	STEC-HUS	Primary and/or secondary care	< 5	< 7.7	12.0	41.4	< 5	< 2.0	11.3	14.0	2.9	0.188
**Cardiac**												
	STEC	Primary care only	50	4.0	12.2	31.5	170	3.5	11.5	28.3	1.1	0.506
		Primary and/or secondary care	127	10.2	11.0	86.2	460	9.4	10.5	82.0	1.1	0.619
	STEC-HUS	Primary care only	< 5	< 7.7	11.3	56.5	< 5	< 2.0	11.3	10.6	5.3	0.038
		Primary and/or secondary care	< 10	< 15.4	10.2	106.3	13	5.1	10.7	47.7	2.2	0.105
**Gastrointestinal**												
	STEC	Primary care only	39	3.1	12.1	24.5	91	1.9	11.5	15.0	1.6	0.014
		Primary and/or secondary care	106	8.5	11.5	69.2	278	5.7	11.3	46.9	1.5	0.001
	STEC-HUS	Primary care only	< 5	< 7.7	11.5	27.8	< 5	< 2.0	11.3	7.0	4.0	0.212
		Primary and/or secondary care	10	15.4	10.4	150.0	< 10	< 3.9	11.3	28.5	5.3	0.001
**Respiratory**												
	STEC	Primary care only	340	27.3	9.4	260.2	1016	20.8	10.0	192.3	1.4	0.000
		Primary and/or secondary care	371	29.8	9.2	288.4	1122	23.0	9.7	214.6	1.3	0.000
	STEC-HUS	Primary care only	20	30.8	8.7	337.2	48	18.8	9.6	192.7	1.7	0.043
		Primary and/or secondary care	< 25	< 38.5	8.5	400.7	53	20.7	9.6	214.7	1.9	0.017
**Endocrine**												
	STEC	Primary and/or secondary care	41	3.3	12.1	25.8	177	3.6	11.4	29.6	0.9	0.435
	STEC-HUS	Primary and/or secondary care	< 5	< 7.7	11.5	27.4	< 5	< 2.0	11.3	10.5	2.6	0.330

^1^Median

Considering the first outcome recorded in primary and/or secondary care, the median time to event was as follows, ranked first to last: 3.4 years (IQR:1.8–6.8 years) for respiratory, 7.6 years (IQR: 2.9–14 years) for cardiac, 7.7 years (IQR: 3.3–12 years) for gastrointestinal, 8.8 years (IQR: 2.9–15 years) for kidney, 8.9 years (IQR: 4.9–13 years) for neurological, and 9.2 years (IQR: 4.5–14 years) for endocrine outcomes (Table 1). The median time to first outcome recorded in primary care only was as follows, ranked first to last: 4.3 years (IQR: 2.2–8.6 years) for respiratory, 6.2 years (IQR: 4.9–10) for neurological, 7.5 years (2.6–12) for kidney, 9.5 years (IQR: 4.6–14) for cardiac, and 11 years (IQR: 5.7–13) for gastrointestinal outcomes.

#### Unexposed

Among 13,385,586 potentially eligible individuals, 4,874 were included in the STEC O157 analysis (Fig. 1). Unexposed individuals were more likely to have no co-morbidities (95% vs. 91%, *p* < 0.001), live in an area of greater deprivation (23% vs. 16%, *p* < 0.001), not seek hospital care during their equivalent’s acute episode (2.5% vs. 32%, *p* < 0.001), and emigrate (16% vs. 8.3%, *p* < 0.001) compared to their exposed counterparts (Table 1).

The incidence of respiratory outcomes managed only recorded in primary care (IRR: 1.4, *p* < 0.001) and primary and/or secondary care (IRR: 1.3, *p* < 0.001) was higher in the exposed compared to the unexposed (Table 2). The incidence of gastrointestinal outcomes was also higher in the exposed (IRR primary care only: 1.6, *p* = 0.01, IRR primary and/or secondary care: 1.5, *p* = 0.001). The median time to first respiratory outcome among individuals only recorded in primary care only was longer in the unexposed (5.3 vs. 4.3 years, *p* = 0.004, see Table 1).

#### Univariate

**Fig. 2:**
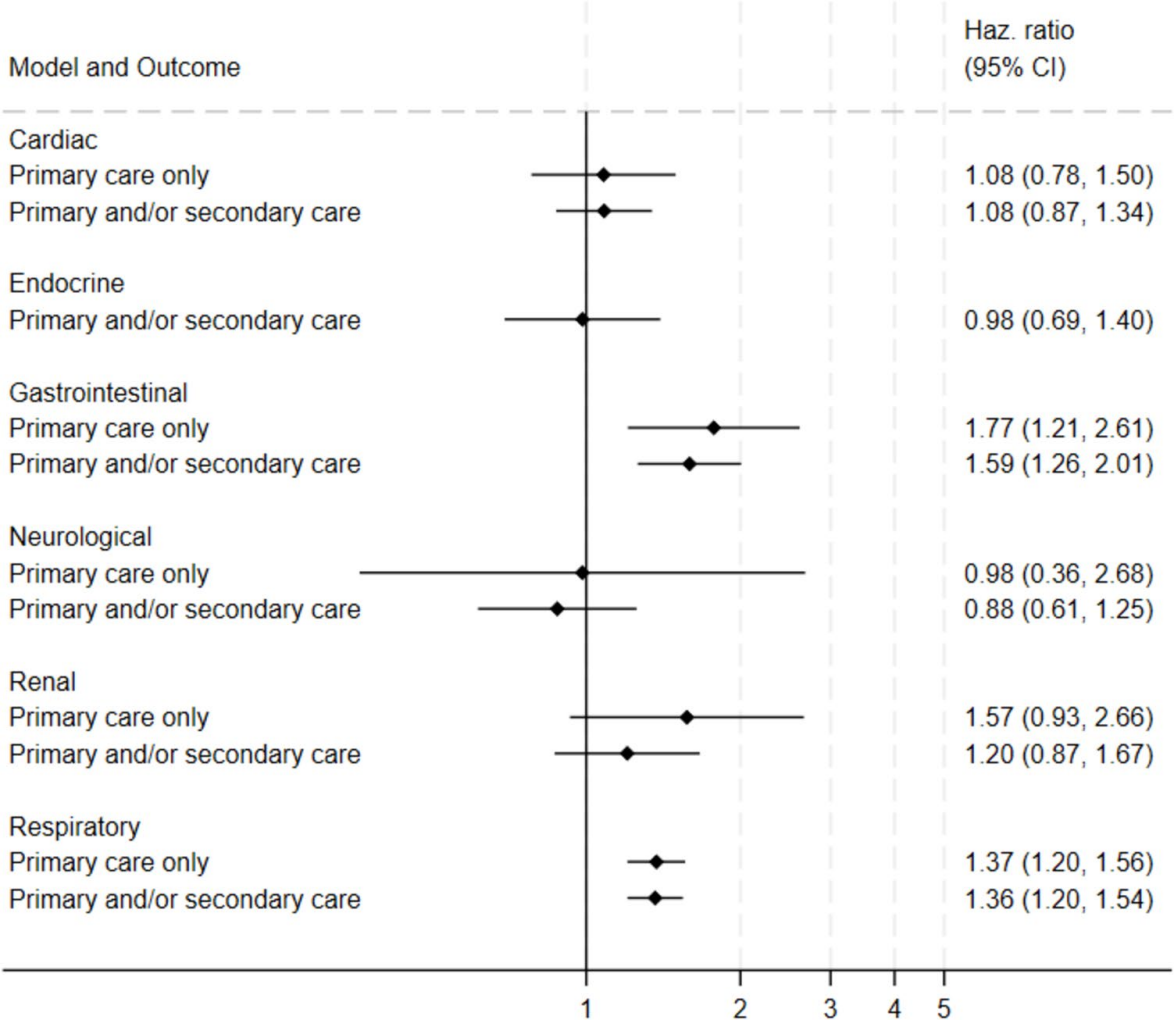
Forest plot showing crude hazard ratios (HR) and 95% confidence intervals for outcomes (cardiac, gastrointestinal, neurological, kidney, respiratory, endocrine) among individuals exposed to Shiga toxin-producing *Escherichia coli* O157 infection (STEC O157), Wales, 1990–2020. Outcomes were defined using clinical code lists for General Practitioner practice consultations ("Primary care only") and/or hospital admissions ("Primary and/or secondary care"). Unexposed cohorts were individuals registered with a General Practitioner practice in Wales who were the same age and sex as their exposed equivalent at time of STEC O157 specimen or symptom onset date

Gastrointestinal outcomes were more common in those exposed to STEC O157 compared to their unexposed equivalents (HR for primary care only: 1.8, 95% CI: 1.2–2.6; HR for primary and/or secondary care: 1.6, 95% CI: 1.3–2.0) (Fig. 2, Supplementary Appendix E-K). Respiratory outcomes were also more common in the exposed (HR for primary care only: 1.4, 95% CI: 1.2–1.6; HR for primary and/or secondary care: 1.4, 95% CI: 1.2–1.5).

#### Multivariable

**Fig. 3:**
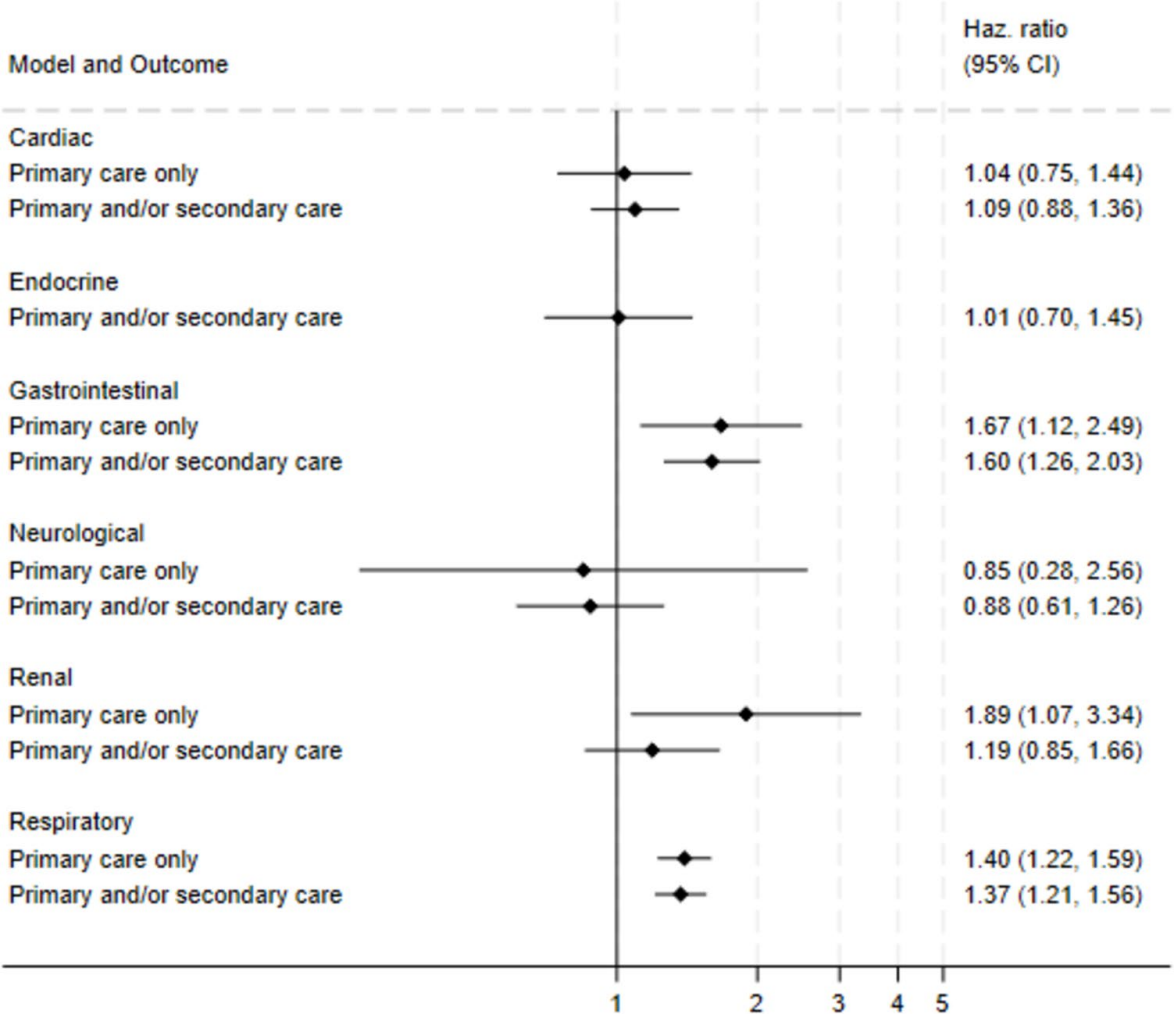
Forest plot showing adjusted hazard ratios (HR) and 95% confidence intervals for outcomes (cardiac, gastrointestinal, neurological, kidney, respiratory, endocrine) among individuals exposed to Shiga toxin-producing *Escherichia coli* O157 infection (STEC O157), Wales, 1990–2020. Estimates adjusted for Welsh Index of Multiple Deprivation and co-morbidities at baseline. Outcomes were defined using clinical code lists for General Practitioner practice consultations ("Primary care only") and/or hospital admissions ("Primary and/or secondary care"). Unexposed cohorts were individuals registered with a General Practitioner practice in Wales who were the same age and sex as their exposed equivalent at time of STEC O157 specimen or symptom onset date

After adjusting for WIMD and co-morbidities, gastrointestinal outcomes were more common in those exposed to STEC O157 compared to the unexposed (aHR for primary care only: 1.7, 95% CI: 1.1–2.5; aHR for primary and/or secondary care: 1.6, 95% CI: 1.3–2.0) (Fig. 3, Supplementary Appendix L). Kidney outcomes managed in primary care only were more common in the exposed (aHR: 1.9, 95% CI: 1.1–3.3), as well all respiratory outcomes (aHR: 1.4, 95% CI: 1.2–1.6).

### STEC-HUS

#### Exposed

Among 1,245 individuals with STEC O157, 65 (5.2%) were reported to develop HUS and were included in the STEC-HUS analysis (Fig. 1). The median age was 5 years (IQR: 2–10 years) (Table 3). The majority were female (48/65, 74%). All sought hospital care in the acute episode. At ≥ 1 year after study entry, there were five (7.7%) individuals who were reported to have died or emigrated (Table 3).

**Table 3:** Characteristics of study participants. Exposure was to Shiga toxin-producing *Escherichia coli* O157 infection (STEC O157)-associated haemolytic uraemic syndrome (HUS), Wales, 1990–2020. The unexposed cohort comprised individuals registered with a General Practitioner practice in Wales who were the same age and sex as their exposed equivalent at time of STEC O157 specimen or symptom onset date

	Study group		
	Unexposed, *N* = 256^1^	Exposed, *N* = 65^1^	*p*-value^2^
**Study entry**			> 0.9
January-June	94 (37%)	24 (37%)	
July-December	162 (63%)	41 (63%)	
**Age (years)**	5 (2, 10)	5 (2, 10)	> 0.9
**Gender**			> 0.9
Male	66 (26%)	17 (26%)	
Female	190 (74%)	48 (74%)	
**Index of multiple deprivation**			0.093
Q5 (least deprived)	44 (17%)	10 (15%)	
Q4	55 (21%)	13 (20%)	
Q3	51 (20%)	16 (25%)	
Q2	42 (16%)	18 (28%)	
Q1 (most deprived)	64 (25%)	8 (12%)	
**Co-morbidity score**			0.011
Zero	249 (97%)	58 (89%)	
One or more	7 (3%)	7 (11%)	
**Sought care in acute episode**	8 (3.1%)	65 (100%)	< 0.001
**Died**	7 (2.7%)	5 (7.7%)	0.072
**Emigrated**	38 (15%)	5 (7.7%)	0.13
**Kidney**			
Primary care only			
Outcome	< 5 (< 2.0%)	< 5 (< 7.7%)	0.3
Time to outcome (years)	6.4 (6.0, 6.5)	4.5 (3.6, 5.4)	0.4
Primary and/or secondary care			
Outcome	< 10 (< 3.9%)	< 10 (< 15.4%)	0.006
Time to outcome (years)	6.9 (6.5, 9.9)	2.7 (2.0, 9.7)	0.2
**Neurological**			
Primary and/or secondary care			
Outcome	< 5 (< 2.0%)	< 5 (< 7.7%)	0.2
Time to outcome (years)	12.3 (11.6, 13.9)	13.1 (9.2, 13.6)	> 0.9
**Cardiac**			
Primary care only			
Outcome	< 5 (< 2.0%)	< 5 (< 7.7%)	0.033
Time to outcome (years)	2.1 (1.6, 3.3)	4.8 (2.9, 7.2)	0.4
Primary and/or secondary care			
Outcome	13 (5.1%)	< 10 (< 15.4%)	0.14
Time to outcome (years)	7.2 (3.9, 11.8)	3.4 (1.8, 5.7)	0.11
**Gastrointestinal**			
Primary care only			
Outcome	< 5 (< 2.0%)	< 5 (< 7.7%)	0.2
Time to outcome (years)	5.2 (4.9, 5.5)	7.7 (4.7, 10.8)	> 0.9
Primary and/or secondary care			
Outcome	< 10 (< 3.9%)	10 (15%)	< 0.001
Time to outcome (years)	5.2 (2.0, 11.3)	3.1 (1.8, 8.6)	0.5
**Respiratory**			
Primary care only			
Outcome	48 (19%)	20 (31%)	0.034
Time to outcome (years)	4.1 (1.9, 8.3)	3.8 (2.0, 6.0)	0.6
Primary and/or secondary care			
Outcome	53 (21%)	< 25 (< 38.5%)	0.013
Time to outcome (years)	4.0 (1.6, 7.6)	4.2 (1.9, 6.1)	0.9
**Endocrine**			
Primary and/or secondary care			
Outcome	< 5 (< 2.0%)	< 5 (< 7.7%)	0.3
Time to outcome (years)	8.1 (6.1, 10.9)	10.5 (9.5, 11.4)	0.8

^1^*n* (%); Median(IQR)

^2^Pearson’s Chi-squared test; Wilcoxon rank sum test; Fisher’s exact test; Wilcoxon rank sum exact test

The median follow-up time is reported for each model in Table 2, ranging from 8.5–12 years. Considering the first outcome recorded in primary and/or secondary care ≥ 1 year after study entry, the incidence of respiratory outcomes was the highest (401 per 10,000 person-years at risk), followed by gastrointestinal (150 per 10,000 person-years at risk), cardiac (106 per 10,000 person-years at risk), kidney (103 per 10,000 person-years at risk), neurological (41 per 10,000 person-years at risk) and endocrine (27 per 10,000 person-years at risk) outcomes. These included acute respiratory tract infection, inflammatory bowel disease, hypertension, and kidney failure. The incidence of outcomes among individuals who were only recorded in primary care was lower, as follows: respiratory (337 per 10,000 person-years), cardiac (57 per 10,000 person-years at risk), kidney (28 per 10,000 person-years at risk), and gastrointestinal (28 per 10,000 person-years at risk). Kidney outcomes among individuals only reported in primary care tended to be earlier stages of kidney disease.

Considering the first outcome recorded in primary and/or secondary care, the median time to event was as follows, ranked first to last: 2.7 years (IQR: 2.0–9.7 years) for kidney, 3.1 years (IQR: 1.8–8.6 years) for gastrointestinal, 3.4 years (IQR: 1.8–5.7 years) for cardiac, 4.2 years (IQR: 1.9–6.1 years) for respiratory, 11 years (IQR: 9.5–11 years) for endocrine, and 13 years (IQR: 9.2–14 years) for neurological outcomes (Table 3). The median time to first outcome recorded in primary care only was as follows, ranked first to last: 3.8 years (IQR: 2.0–6.0 years) for respiratory, 4.5 years (IQR: 3.6–5.4) for kidney, 4.8 years (2.9–7.2) for cardiac, and 7.7 years (IQR: 4.7–11) for gastrointestinal outcomes.

#### Unexposed

There were 256 unexposed individuals included in the STEC-HUS analysis (Fig. 1). Unexposed individuals were more likely to have no co-morbidities (97% vs. 89%, *p* = 0.011) and not seek hospital care during their equivalent’s acute episode (3.1% vs. 100%, *p* < 0.001) compared to their exposed counterparts (Table 3).

The incidence rate of gastrointestinal (IRR: 5.3, *p* < 0.001), kidney (IRR: 4.9, *p* = 0.007) and respiratory (IRR: 1.9, *p* = 0.02) outcomes among individuals recorded in primary and/or secondary care was higher in the exposed compared to the unexposed (Table 2). The incidence rate of cardiac (IRR: 5.3, *p* = 0.04) and respiratory (IRR: 1.7, *p* = 0.04) outcomes among individuals only recorded in primary care was also greater in the exposed. No difference in median time to first outcome was observed between the exposed and unexposed (Table 3).

#### Univariate

**Fig. 4:**
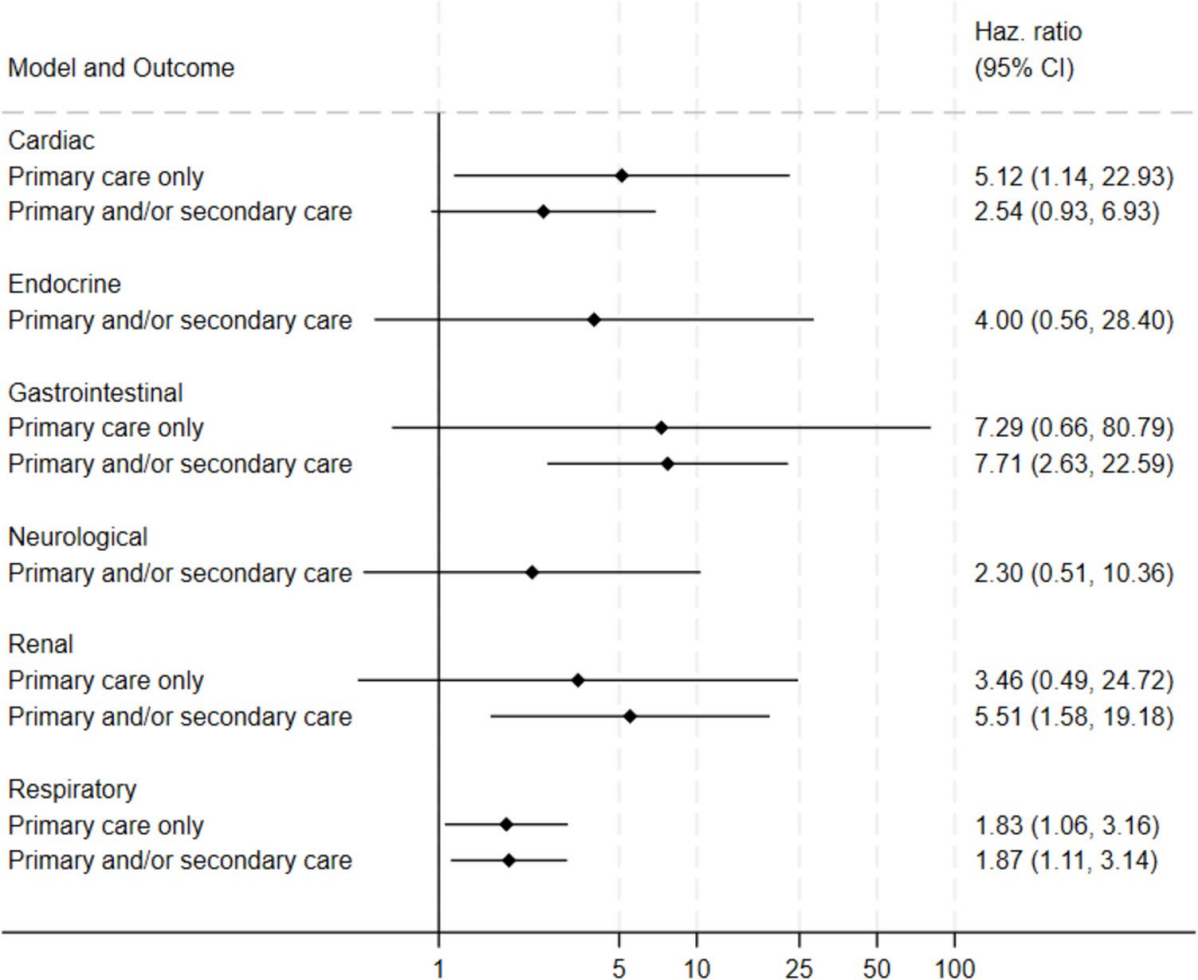
Forest plot showing crude hazard ratios (HR) and 95% confidence intervals for outcomes (cardiac, gastrointestinal, neurological, kidney, respiratory, endocrine) among individuals exposed to Shiga toxin-producing *Escherichia coli* O157 infection (STEC O157) with haemolytic uraemic syndrome (HUS), Wales, 1990–2020. Outcomes were defined using clinical code lists for General Practitioner practice consultations ("Primary care only") and/or hospital admissions ("Primary and/or secondary care"). Unexposed cohorts were individuals registered with a General Practitioner practice in Wales who were the same age and sex as their exposed equivalent at time of STEC O157 specimen or symptom onset date

Gastrointestinal outcomes were more common in those exposed to STEC-HUS compared to their unexposed equivalents (HR for primary and/or secondary care: 7.7, 95% CI: 2.6–23) (Fig. 4, Supplementary Appendix E-K). Differences were also observed for the following outcomes, ranked highest to lowest on effect size: kidney (HR for primary and/or secondary care: 5.5, 95% CI: 1.6–19), cardiac (HR for primary care only: 5.1, 95% CI: 1.1–23), and respiratory (HR for primary and/or secondary care: 1.9, 95% CI: 1.1–3.1; HR for primary care only: 1.8, 95% CI: 1.1–3.2).

## Discussion

We identified prominent long-term health complications in a subset of individuals following STEC O157. Complications were more common and occurred sooner after infection in those who developed HUS, most of whom were children at the time of their exposure. Nevertheless, there was no record of long-term complications for the majority and no difference in the proportion who died after the first year following infection. Where complications were observed, the greatest relative risk was among those exposed to STEC-HUS, who were eight times more likely than their unexposed counterparts to experience gastrointestinal outcomes. This included conditions such as inflammatory bowel disease, which were first recorded on average three years after exposure. Kidney, cardiac, and respiratory complications were also relatively more likely in this group, ranging from six- to two-fold greater, and tended to be first recorded between three to five years after exposure. The greatest relative risk for those with STEC O157 was kidney complications, who were nearly twice as likely as their unexposed counterparts to seek care for conditions such as chronic kidney disease. Gastrointestinal and respiratory complications were also more common in this group, with the latter occurring on average one year sooner than in the unexposed. Contrasting to those who developed HUS, individuals in the STEC O157 cohort tended to be older at the time of their exposure and experience outcomes several years later. These findings underscore the potential harmful long-term consequences in those exposed to STEC O157, whether they had HUS during the acute episode or not. Short-term follow-up that is limited to well-documented outcomes, such as complications involving the kidneys and central nervous system, or to exposure during childhood, may therefore underestimate the extent and severity of complications of STEC O157.

It is well-established that devastating kidney complications can be observed in those with a history of STEC resulting in HUS. In a meta-analysis of 49 studies with mean follow-up of 4.4 years, death or permanent kidney failure occurred in approximately 12% of those with diarrhoea-associated HUS [11]. In a study with ≥ 10 years follow-up after paediatric diarrhoea-associated HUS, serious kidney complications were observed in 9.4% [34]. Our estimates for long-term kidney complications managed in primary care only were lower, under 8%. However, outcomes in this model more commonly included milder forms of disease, such as earlier stages of chronic kidney disease. When we included secondary care data in the model, a proxy measure for more severe outcomes, our estimates were more like those in the meta-analysis and were statistically significant.

The impact of STEC without HUS on long-term kidney function has been less explored, likely because the acute phase is often self-limiting in adults and may therefore not result in healthcare-seeking behaviour [35]. One cohort study that did investigate this found an association between STEC O157 and *Campylobacter* in adulthood and long-term kidney impairment [35, 36], but no effect after restricting the study population to children [37]. The authors speculated that the differences were because children were more likely to be diagnosed, treated, and recover, and adults were more likely to have co-morbidities that could confound the association. After adjusting for the effect of deprivation and pre-existing co-morbidities, we observed a two-fold increased risk of kidney complications among those with STEC O157, but only for complications managed solely in primary care. No statistically significant association was observed for complications also managed in secondary care, which more commonly included severe outcomes like kidney failure.

Studies of extrarenal complications of STEC have tended to focus on individuals who also had HUS. Those describing long-term gastrointestinal sequelae after HUS reported estimates comparable to our model of gastrointestinal outcomes among individuals attending primary and/or secondary care [38, 39]. We provide evidence that STEC O157 might also, in the absence of HUS, cause gastrointestinal harm several years after acute infection. Although Shiga toxin-mediated pathogenesis is not fully understood, these toxins are known to induce proinflammatory responses, exacerbate tissue damage, and contribute to dysbiosis in the gastrointestinal tract [12, 13, 40]. These are associated with a variety of disorders including inflammatory bowel disease [40], which was more frequently identified in those following STEC O157 in our study compared to the unexposed group.

While neurological involvement is the most life-threatening acute complication of STEC-HUS [12], we observed no difference in the incidence of long-term neurologic sequelae in survivors of STEC O157, with and without HUS, compared to their unexposed equivalents. This is consistent with one of the largest cohort studies which reported complete neurologic recovery in most STEC-HUS patients [41], as well as a case control study which found no subclinical effects on IQ, behaviour, verbal abilities, or academic achievement [42]. We also found no difference in the likelihood of endocrine outcomes, namely diabetes, in those exposed to STEC O157 or STEC-HUS.

We observed mixed results for the other extrarenal complications assessed in this study. Cardiovascular disease, namely hypertension, that was managed in primary care only was over five times more likely following STEC-HUS compared to the unexposed group, but no difference was seen in those with STEC O157. One of the few other studies of long-term cardiac outcomes did find an increased risk of myocardial infarctions, stroke, and congestive heart failure if STEC O157 or *Campylobacter* was acquired during adulthood [35, 36], but not of its precursors, such as hypertension, in children [37]. Conversely, respiratory complications, namely acute respiratory tract infections, were more frequently observed in our study following STEC-O157 and STEC-HUS. Severe respiratory complications, such as pulmonary collapse and chronic respiratory failure, have been reported elsewhere during the acute phase of STEC-HUS [43], but were not assessed in this study and, like cardiac complications, remain largely unexplored in the long-term. Further investigations are therefore required to clarify the potential risk of respiratory and cardiovascular disease as extrarenal complications of STEC O157 with or without HUS.

While it is a statutory duty to report STEC O157 and HUS under the Health Protection (Notification) Regulations (2010) [44], STEC is a self-limiting illness in most cases, so many may never seek healthcare. As deprivation is associated with reduced access to healthcare [45], it is possible that infections were more likely to be missed in the STEC O157 unexposed cohort, among whom greater rates of deprivation were observed. It is estimated that even in those with HUS, for which hospitalisation is almost certain, two thirds may not be confirmed to be associated with STEC [46]. However, as STEC is a rare exposure, it is unlikely that many of the unexposed were misclassified. Regarding STEC-HUS, we identified HUS in 5.2% of those with STEC O157, like estimates reported elsewhere [5, 10]. Nevertheless, despite using 30 years of routine surveillance data, sample size for STEC-HUS analyses was limited. We were therefore likely underpowered to detect associations in HUS analyses and could not adjust for confounding by deprivation or co-morbidities in this group, which may have inflated hazard ratios.

Prior to 2013, PCR testing was not widely available in the UK for gastrointestinal screening for faecal samples [5]. As differentiation of STEC O157 from non-O157 serogroups relies on PCR testing, it is possible we included individuals with non-O157 STEC in our study. The proportion potentially misclassified is not quantifiable since the true incidence of non-O157 remains unknown [5]. However, it is increasingly recognised that non-O157 STEC can cause severe, even fatal, outcomes [47, 48], and that Shiga toxin and *E. coli* attachment and effacing genes, as opposed to serogroup, are more indicative of potential virulence [49]. With growing focus on STEC genotype, the possible inclusion of non-O157 STEC infections in our study might therefore be of little clinical significance.

All hospitals in Wales share data with the SAIL Databank, as do approximately 80% of GP practices [50]. Individuals who were not registered with a GP practice or who were registered with a practice that did not share data with the SAIL Databank would therefore have been excluded from our study. Additionally, complications managed in primary care only may have been under-ascertained, which could have weakened associations with the exposures. Mild complications, such as some forms of cognitive impairment, were also more likely to be managed without healthcare consultation, which could have compounded our inability to detect associations.

Unexposed individuals were more likely to emigrate compared to their equivalents with STEC O157. As there was differential follow-up based on exposure status, it is possible that there was a selection bias for detection of poor health outcomes in those with infection. Finally, both unexposed cohorts had fewer co-morbidities at baseline. However, co-morbidities were largely uncommon in the exposed groups. We also chose to assess acute or new chronic conditions after study entry to minimise the impact of pre-existing ill health on the outcomes of interest.

## Conclusions

For most individuals exposed to STEC O157, no complications were observed more than one year after the acute phase of infection, regardless of the occurrence of HUS. Similarly, those that survived the first year following infection were at no greater risk of death compared to general population comparators. Nevertheless, long-term complications were nearly twice as likely in those exposed to STEC O157, and as many as eight times more likely in those with STEC-HUS. This included potentially life changing conditions such as inflammatory bowel disease and chronic kidney disease. In some instances, these were newly reported over a decade after infection. Our findings are therefore supportive of a protracted, multi-organ burden of disease associated with STEC O157. As the clearest risk was for infections that resulted in HUS, early interventions that aim to prevent this are likely crucial for altering the long-term trajectory of disease. Even in the absence of HUS, we demonstrate that short-term follow-up that is limited to well-documented outcomes, such as complications involving the kidneys and central nervous system, or to exposure during childhood, may underestimate the extent and severity of complications of STEC O157. Lifelong follow-up has already been recommended by some after STEC-HUS [14], and we suggest that follow-up periods of at least five years also be considered for infections which do not progress to HUS. Additionally, we recommend the scope of potential extrarenal sequelae be expanded to include gastrointestinal, respiratory, and cardiac complications. Finally, with the increasing availability of genetic sequencing information, cohort studies of so-called high-risk subtypes [49] should be conducted to comment on the suitability of extrapolating our findings to non-O157 STEC.

## Supplementary Information

Below is the link to the electronic supplementary material.

Graphical abstract (PPTX 170 KB)

Supplementary file2 (DOCX 588 KB)

## Data Availability

The data used in this study are available in the SAIL Databank at Swansea University, Swansea, UK. As restrictions apply, data are only available to bona fide researchers. All proposals to use SAIL data are subject to review by an independent Information Governance Review Panel (IGRP). Before any data can be accessed, approval must be given by the IGRP. The IGRP gives careful consideration to each project to ensure proper and appropriate use of SAIL data. When access has been granted, it is gained through a privacy protecting haven and remote access system, referred to as the SAIL Gateway. SAIL has established an application process to be followed by anyone who would like to access data via SAIL at https://saildatabank.com/data/apply-to-work-with-the-data/.
