# Supplementary material for: Long-term health outcomes of Shiga toxin-producing Escherichia coli O157 (STEC O157) infection and STEC-associated haemolytic uraemic syndrome (STEC-HUS), Wales, 1990–2020
Source: Pediatr Nephrol. 2025 Feb 4;40(7):2295–310. doi: 10.1007/s00467-024-06640-x (PMC12116988; doi:10.1007/s00467-024-06640-x)
Supplement: Supplementary file 1 — Graphical abstract (PPTX 170 KB) [file 467_2024_6640_MOESM1_ESM.pptx]

## Slide 1
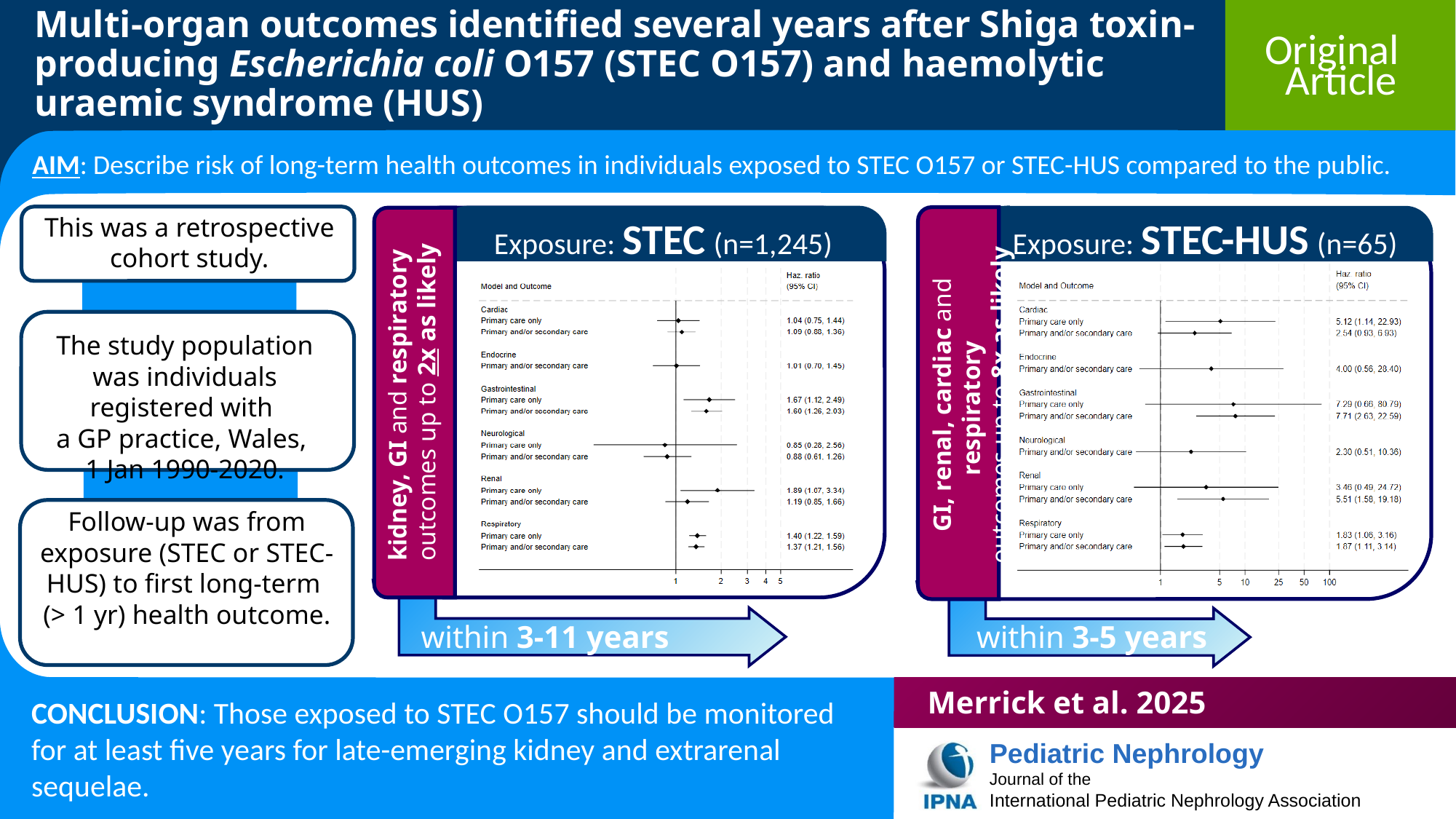

Multi-organ outcomes identified several years after Shiga toxin-producing Escherichia coli O157 (STEC O157) and haemolytic uraemic syndrome (HUS)
AIM: Describe risk of long-term health outcomes in individuals exposed to STEC O157 or STEC-HUS compared to the public.
This was a retrospective cohort study.
Exposure: STEC (n=1,245)
Exposure: STEC-HUS (n=65)
The study population was individuals registered with
a GP practice, Wales,
1 Jan 1990-2020.
kidney, GI and respiratory
outcomes up to 2x as likely
GI, renal, cardiac and respiratory
outcomes up to 8x as likely
Follow-up was from exposure (STEC or STEC-HUS) to first long-term
(> 1 yr) health outcome.
within 3-11 years
within 3-5 years
Merrick et al. 2025
CONCLUSION: Those exposed to STEC O157 should be monitored for at least five years for late-emerging kidney and extrarenal sequelae.
