# Supplementary material for: Long-term health outcomes of Shiga toxin-producing Escherichia coli O157 (STEC O157) infection and STEC-associated haemolytic uraemic syndrome (STEC-HUS), Wales, 1990–2020
Source: Pediatr Nephrol. 2025 Feb 4;40(7):2295–310. doi: 10.1007/s00467-024-06640-x (PMC12116988; doi:10.1007/s00467-024-06640-x)
Supplement: Supplementary file 2 — Supplementary file2 (DOCX 588 KB) [file 467_2024_6640_MOESM2_ESM.docx]

**Supplementary Information**

Article title: Long-term health outcomes of Shiga toxin-producing *Escherichia coli* O157 (STEC O157) infection and STEC-associated haemolytic uraemic syndrome (STEC-HUS), Wales, 1990-2020

Journal name: Pediatric Nephrology

Author names: Rachel Merrick^1^, Jiao Song^1^, Laia Fina^1^, Clare Sawyer^1^, Claire Jenkins^2^, Grace King^2^, Drew Turner^1^, Daniel Thomas^1^, Christopher Williams^1^

Affiliations:

1. Public Health Wales, Cardiff, United Kingdom
2. UK Health Security Agency, London, United Kingdom

Corresponding author: [christopher.williams25@wales.nhs.uk](mailto:christopher.williams25@wales.nhs.uk)

**APPENDICES**

**Appendix A: ICD-10 codes for health outcomes identified in secondary care**

| **Outcome** | **ICD-10 code** |
| --- | --- |
| **Kidney** | |
| Acute renal failure | N17 |
| Unspecified kidney failure | N19 |
| Chronic kidney disease | N18 |
| Kidney transplant status | Z940 |
| Kidney dialysis | Y841 |
| Kidney transplant failure and rejection | T861 |
| Chronic nephritic syndrome | N03 |
| Disorders resulting from impaired renal tubular function | N25 |
| Dependence on renal dialysis | Z992 |
| Unspecified nephritic syndrome | N05 |
| Rapidly progressive nephritic syndrome | N01 |
| Acute nephritic syndrome | N00 |
| **Neurological** | |
| Epilepsy | G40 |
| Status epilepticus | G41 |
| Somnolence, stupor, and coma | R40 |
| Altered cognitive function and awareness | R41 |
| Dizziness and giddiness | R42 |
| Altered sensations and perceptions | R44 |
| Altered emotional state | R45 |
| Altered appearances and behaviour | R46 |
| **Cardiac** | |
| Angina pectoris | I20 |
| Acute myocardial infarction | I21 |
| Subsequent myocardial infarction | I22 |
| Certain current complications following acute myocardial infarction | I23 |
| Other acute ischaemic heart diseases | I24 |
| Chronic ischaemic heart disease | I25 |
| Primary hypertension | I10 |
| Hypertensive heart disease | I11 |
| Hypertensive renal disease | I12 |
| Hypertensive heart and renal disease | I13 |
| Secondary hypertension | I15 |
| **Gastrointestinal** | |
| Irritable bowel syndrome | K58 |
| Arthropathy in ulcerative colitis | M075 |
| Arthropathy in Crohn’s disease [regional enteritis] | M074 |
| Other functional intestinal disorders | K59 |
| Crohn’s disease | K50 |
| Ulcerative colitis | K51 |
| Other noninfective gastroenteritis and colitis | K52 |
| **Respiratory** | |
| Acute upper respiratory infections of multiple and unspecified sites | J06 |
| Unspecified acute lower respiratory infection | J22 |
| Pneumonia, organism unspecified | J18 |

**Appendix B: Read (v2) codes for health outcomes identified in primary care [Ref: 19-27]**

| **Outcome** | **Read (v2) code** |
| --- | --- |
| **Kidney** (end stage kidney disease) | |
| peritoneal dialysis | 7L1A100 |
| acquired arteriovenous fistula | G760.00 |
| haemodialysis nec | 7L1A200 |
| transplantation of kidney | 7B00.00 |
| creation of arteriovenous fistula nec | 7A60100 |
| transplantation of kidney nos | 7B00z00 |
| [v]kidney transplanted | ZV42000 |
| end stage renal failure | K050.00 |
| insertion of ambulatory peritoneal dialysis catheter | 7L1B000 |
| end-stage renal disease | K0D..00 |
| ligation of acquired arteriovenous fistula | 7A61400 |
| kidney transplant failure and rejection | SP08300 |
| transplantation of kidney from live donor | 7B00100 |
| dialysis for renal failure | 7L1A.11 |
| chronic kidney disease stage 5 | 1Z14.00 |
| renal transplant planned | 8L50.00 |
| renal transplant with complication, without blame | TB00111 |
| repair of acquired arteriovenous fistula | 7A61100 |
| renal dialysis | 7L1A000 |
| h/o: renal dialysis | 14V2.00 |
| [v]renal dialysis status | ZV45100 |
| removal of ambulatory peritoneal dialysis catheter | 7L1B100 |
| arteriovenous shunt | 7A60.00 |
| transplantation of kidney from cadaver | 7B00200 |
| creation of brachial-cephalic fistula | 7A60112 |
| exploration of renal transplant | 7B06300 |
| kidney dialysis with complication, without blame | TB11.00 |
| creation of radial-cephalic fistula | 7A60111 |
| insertion of temporary peritoneal dialysis catheter | 7L1C000 |
| continuous ambulatory peritoneal dialysis | 7L1A500 |
| removal of infected arteriovenous shunt | 7A60300 |
| compensation for renal failure | 7L1A.00 |
| failure of sterile precautions during perfusion | TA22.00 |
| placement ambulatory dialysis apparatus - compens renal fail | 7L1B.11 |
| h/o: kidney dialysis | 14V2.11 |
| [v]aftercare involving peritoneal dialysis | ZV56y11 |
| [v]aftercare involving renal dialysis nos | ZV56011 |
| [x] peritoneal dialysis associated peritonitis | SP05613 |
| other specified compensation for renal failure | 7L1Ay00 |
| renal tubulo-interstitial disordrs in transplant rejectn | K0B5.00 |
| transplant nephrectomy | 7B01500 |
| mechanical complication of dialysis catheter | SP01500 |
| mechanical complication of arterio-venous surgical fistula | SP01700 |
| h/o: kidney recipient | 14S2.00 |
| [v]preparatory care for dialysis | ZV56100 |
| end stage renal failure | K05..12 |
| [x]failure sterile precautions dur kidney dialys/other perf | U612200 |
| kidney transplant with complication, without blame | TB00100 |
| placement ambulatory apparatus compensation renal failure | 7L1B.00 |
| arteriovenous shunt nos | 7A60z00 |
| placement ambulatory apparatus- compensate renal failure os | 7L1By00 |
| stenosis of arteriovenous dialysis fistula | SP07G00 |
| creation of graft fistula for dialysis | 7A60600 |
| care of haemodialysis equipment | Z919.00 |
| reversing haemodialysis lines | Z919300 |
| [v]aftercare involving intermittent dialysis | ZV56.00 |
| insertion of arteriovenous prosthesis | 7A60000 |
| attention to arteriovenous shunt | 7A60200 |
| thrombectomy of arteriovenous fistula | 7A60500 |
| [v]other specified aftercare involving intermittent dialysis | ZV56y00 |
| peritoneal dialysis bag procedure | Z91A.00 |
| compensation for renal failure nos | 7L1Az00 |
| peritoneal dialysis nec | 7L1A600 |
| placement other apparatus- compensate for renal failure nos | 7L1Cz00 |
| other specified arteriovenous shunt | 7A60y00 |
| allotransplantation of kidney from live donor | 7B00111 |
| renal dialysis with complication, without blame | TB11.11 |
| failure of sterile precautions during kidney dialysis | TA22000 |
| [x]other dialysis | ZVu3G00 |
| det.ren.func.after ren.transpl | SP08011 |
| other specified transplantation of kidney | 7B00y00 |
| haemofiltration | 7L1A300 |
| excision of rejected transplanted kidney | 7B01511 |
| priming haemodialysis lines | Z919100 |
| haemodialysis training | Z1A2.00 |
| placement other apparatus for compensation for renal failure | 7L1C.00 |
| dialysis fluid glucose level | 4N2..00 |
| automated peritoneal dialysis | 7L1A400 |
| predicted stage chronic kidney disease | 9Ot5.00 |
| allotransplantation of kidney from cadaver heart-beating | 7B00300 |
| pre-transplantation of kidney work-up, recipient | 7B0F100 |
| interventions associated with transplantation of kidney | 7B0F.00 |
| chronic kidney disease stage 5 without proteinuria | 1Z1L.00 |
| chronic kidney disease stage 5 with proteinuria | 1Z1K.00 |
| banding of arteriovenous fistula | 7A60400 |
| allotransplantation kidney from cadaver heart non-beating | 7B00400 |
| accid cut,puncture,perf,h'ge - kidney dialysis | TA02000 |
| ligation of arteriovenous dialysis fistula | 7A61900 |
| ckd stage 5 without proteinuria | 1Z1L.11 |
| allotransplantation of kidney from cadaver | 7B00211 |
| peritoneal dialysis sample | 4I29.00 |
| ckd stage 5 with proteinuria | 1Z1K.11 |
| [v]aftercare involving extracorporeal dialysis | ZV56000 |
| ligation of acquired arteriovenous fistula | 7A61111 |
| [x]renal tubulo-interstitial disorders/transplant rejection | Kyu1C00 |
| continuous ambulatory peritoneal dialysis associated perit | SP06B00 |
| dialysis fluid urea level | 4N0..00 |
| thomas intravascular shunt for dialysis | 7L1A011 |
| dialysis training | Z1A..00 |
| post-transplantation of kidney examination, recipient | 7B0F300 |
| transplantation surgery | 9b8K.00 |
| interventions associated with transplantation of kidney nos | 7B0Fz00 |
| os interventions associated with transplantation of kidney | 7B0Fy00 |
| flushing of peritoneal dialysis catheter | 7L1B200 |
| peritoneal dialysis training | Z1A1.00 |
| chronic kidney disease stage 5 | K055.00 |
| cadaveric renal transplant | 7B00212 |
| anaphylactoid reaction due to haemodialysis | SP0G.00 |
| unexplained episode of renal transplant dysfunction | SP08N00 |
| aneurysm of anastomotic site of dialysis av fistula | G72D200 |
| ruptured aneurysm of dialysis vascular access | G72C.00 |
| xenograft renal transplant | 7B00600 |
| renal transplant rejection | SP08R00 |
| chronic rejection of renal transplant | SP08J00 |
| thrombosis of dialysis arteriovenous fistula | Gy21.00 |
| vascular complication of renal transplant | SP08W00 |
| haemorrhage of dialysis arteriovenous fistula | Gy51.00 |
| occlusion of dialysis arteriovenous fistula | Gy31.00 |
| discussion about kidney transplantation | 67P4100 |
| aneurysm of dialysis arteriovenous fistula | G72D.00 |
| arteriovenous fistula thrombosis | SP07N00 |
| aneurysm of needle site of dialysis arteriovenous fistula | G72D100 |
| infection of dialysis arteriovenous fistula | Gy41.00 |
| ligation of arteriovenous dialysis graft | 7A61A00 |
| stenosis of dialysis vascular access | Gy1..00 |
| urological complication of renal transplant | SP08T00 |
| disorders associated with peritoneal dialysis | SP0E.00 |
| placement other apparatus- compensate for renal failure os | 7L1Cy00 |
| occlusion of dialysis vascular access | Gy3..00 |
| infection of dialysis arteriovenous graft | Gy40.00 |
| rupture of dialysis arteriovenous graft | Gy60.00 |
| **Kidney** (chronic kidney disease) | |
| H/O: kidney recipient | 14S2.00 |
| Chronic kidney disease stage 1 | 1Z10.00 |
| Chronic kidney disease stage 2 | 1Z11.00 |
| Chronic kidney disease stage 3 | 1Z12.00 |
| Chronic kidney disease stage 4 | 1Z13.00 |
| Chronic kidney disease stage 5 | 1Z14.00 |
| Chronic kidney disease stage 3A | 1Z15.00 |
| Chronic kidney disease stage 3B | 1Z16.00 |
| Chronic kidney disease stage 1 with proteinuria | 1Z17.00 |
| CKD stage 1 with proteinuria | 1Z17.11 |
| Chronic kidney disease stage 1 without proteinuria | 1Z18.00 |
| CKD stage 1 without proteinuria | 1Z18.11 |
| Chronic kidney disease stage 2 with proteinuria | 1Z19.00 |
| CKD stage 2 with proteinuria | 1Z19.11 |
| Chronic kidney disease stage 2 without proteinuria | 1Z1A.00 |
| CKD stage 2 without proteinuria | 1Z1A.11 |
| Chronic kidney disease stage 3 with proteinuria | 1Z1B.00 |
| CKD stage 3 with proteinuria | 1Z1B.11 |
| Chronic kidney disease stage 3 without proteinuria | 1Z1C.00 |
| CKD stage 3 without proteinuria | 1Z1C.11 |
| Chronic kidney disease stage 3A with proteinuria | 1Z1D.00 |
| CKD stage 3A with proteinuria | 1Z1D.11 |
| Chronic kidney disease stage 3A without proteinuria | 1Z1E.00 |
| CKD stage 3A without proteinuria | 1Z1E.11 |
| Chronic kidney disease stage 3B with proteinuria | 1Z1F.00 |
| CKD stage 3B with proteinuria | 1Z1F.11 |
| Chronic kidney disease stage 3B without proteinuria | 1Z1G.00 |
| CKD stage 3B without proteinuria | 1Z1G.11 |
| Chronic kidney disease stage 4 with proteinuria | 1Z1H.00 |
| CKD stage 4 with proteinuria | 1Z1H.11 |
| Chronic kidney disease stage 4 without proteinuria | 1Z1J.00 |
| CKD stage 4 without proteinuria | 1Z1J.11 |
| Chronic kidney disease stage 5 with proteinuria | 1Z1K.00 |
| CKD stage 5 with proteinuria | 1Z1K.11 |
| Chronic kidney disease stage 5 without proteinuria | 1Z1L.00 |
| CKD stage 5 without proteinuria | 1Z1L.11 |
| CKD with GFR category G1 & albuminuria category | 1Z1M.00 |
| CKD with GFR category G1 & albuminuria category | 1Z1N.00 |
| CKD with GFR category G1 & albuminuria category | 1Z1P.00 |
| CKD with GFR category G2 & albuminuria category | 1Z1Q.00 |
| CKD with GFR category G2 & albuminuria category | 1Z1R.00 |
| CKD with GFR category G2 & albuminuria category | 1Z1S.00 |
| CKD with GFR category G3a & albuminuria category | 1Z1T.00 |
| CKD with GFR category G3a & albuminuria category | 1Z1V.00 |
| CKD with GFR category G3a & albuminuria category | 1Z1W.00 |
| CKD with GFR category G3b & albuminuria category | 1Z1X.00 |
| CKD with GFR category G3b & albuminuria category | 1Z1Y.00 |
| CKD with GFR category G3b & albuminuria category | 1Z1Z.00 |
| CKD with GFR category G4 & albuminuria category | 1Z1a.00 |
| CKD with GFR category G4 & albuminuria category | 1Z1b.00 |
| CKD with GFR category G4 & albuminuria category | 1Z1c.00 |
| CKD with GFR category G5 & albuminuria category | 1Z1d.00 |
| CKD with GFR category G5 & albuminuria category | 1Z1e.00 |
| CKD with GFR category G5 & albuminuria category | 1Z1f.00 |
| Chronic kidney disease resolved | 2.13E+03 |
| Arteriovenous shunt | 7A60.00 |
| Insertion of arteriovenous prosthesis | 7A60000 |
| Creation of arteriovenous fistula NEC | 7A60100 |
| Creation of radial-cephalic fistula | 7A60111 |
| Creation of brachial-cephalic fistula | 7A60112 |
| Attention to arteriovenous shunt | 7A60200 |
| Removal of infected arteriovenous shunt | 7A60300 |
| Banding of arteriovenous fistula | 7A60400 |
| Thrombectomy of arteriovenous fistula | 7A60500 |
| Creation of graft fistula for dialysis | 7A60600 |
| Other specified arteriovenous shunt | 7A60y00 |
| Arteriovenous shunt NOS | 7A60z00 |
| Repair of acquired arteriovenous fistula | 7A61100 |
| Ligation of acquired arteriovenous fistula | 7A61111 |
| Ligation of acquired arteriovenous fistula | 7A61400 |
| Ligation of arteriovenous dialysis fistula | 7A61900 |
| Ligation of arteriovenous dialysis graft | 7A61A00 |
| Transplantation of kidney | 7B00.00 |
| Autotransplant of kidney | 7B00000 |
| Transplantation of kidney from live donor | 7B00100 |
| Allotransplantation of kidney from live donor | 7B00111 |
| Transplantation of kidney from cadaver | 7B00200 |
| Allotransplantation of kidney from cadaver | 7B00211 |
| Cadaveric renal transplant | 7B00212 |
| Allotransplantation of kidney from cadaver, heart-beating | 7B00300 |
| Allotransplantation kidney from cadaver, heart non-beating | 7B00400 |
| Allotransplantation of kidney from cadaver NEC | 7B00500 |
| Xenograft renal transplant | 7B00600 |
| Other specified transplantation of kidney | 7B00y00 |
| Transplantation of kidney NOS | 7B00z00 |
| Bilateral nephrectomy | 7B01200 |
| Transplant nephrectomy | 7B01500 |
| Excision of rejected transplanted kidney | 7B01511 |
| Exploration of renal transplant | 7B06300 |
| Interventions associated with transplantation of kidney | 7B0F.00 |
| Pre-transplantation of kidney work-up, recipient | 7B0F100 |
| Post-transplantation of kidney examination, recipient | 7B0F300 |
| OS interventions associated with transplantation of kidney | 7B0Fy00 |
| Interventions associated with transplantation of kidney NOS | 7B0Fz00 |
| Compensation for renal failure | 7L1A.00 |
| Dialysis for renal failure | 7L1A.11 |
| Renal dialysis | 7L1A000 |
| Thomas intravascular shunt for dialysis | 7L1A011 |
| Peritoneal dialysis | 7L1A100 |
| Haemodialysis NEC | 7L1A200 |
| Haemofiltration | 7L1A300 |
| Automated peritoneal dialysis | 7L1A400 |
| Continuous ambulatory peritoneal dialysis | 7L1A500 |
| Peritoneal dialysis NEC | 7L1A600 |
| Other specified compensation for renal failure | 7L1Ay00 |
| Compensation for renal failure NOS | 7L1Az00 |
| Placement ambulatory apparatus compensation renal failure | 7L1B.00 |
| Placement ambulatory dialysis apparatus - compens renal fail | 7L1B.11 |
| Insertion of ambulatory peritoneal dialysis catheter | 7L1B000 |
| Removal of ambulatory peritoneal dialysis catheter | 7L1B100 |
| Flushing of peritoneal dialysis catheter | 7L1B200 |
| Placement ambulatory apparatus- compensate renal failure OS | 7L1By00 |
| Placement other apparatus for compensation for renal failure | 7L1C.00 |
| Insertion of temporary peritoneal dialysis catheter | 7L1C000 |
| Placement other apparatus- compensate for renal failure OS | 7L1Cy00 |
| Placement other apparatus- compensate for renal failure NOS | 7L1Cz00 |
| Extracorporeal albumin haemodialysis | 7L1f000 |
| Renal transplant planned | 8L50.00 |
| Anaemia secondary to renal failure | D215.00 |
| Anaemia secondary to chronic renal failure | D215000 |
| Hypertensive renal disease with renal failure | G222.00 |
| Hypertensive heart and renal disease with renal failure | G233.00 |
| Hyperten heart&renal dis+both(congestv)heart and renal fail | G234.00 |
| Ruptured aneurysm of dialysis vascular access | G72C.00 |
| Aneurysm of dialysis arteriovenous fistula | G72D.00 |
| Aneurysm of superficialised artery of dialysis AV fistula | G72D000 |
| Aneurysm of needle site of dialysis arteriovenous fistula | G72D100 |
| Aneurysm of anastomotic site of dialysis AV fistula | G72D200 |
| Goodpasture's syndrome | G752000 |
| Goodpasture's disease | G752100 |
| Acquired arteriovenous fistula | G760.00 |
| Stenosis of dialysis vascular access | Gy1..00 |
| Stenosis of dialysis arteriovenous graft | Gy10.00 |
| Thrombosis of dialysis vascular access | Gy2..00 |
| Thrombosis of dialysis arteriovenous fistula | Gy21.00 |
| Occlusion of dialysis vascular access | Gy3..00 |
| Occlusion of dialysis arteriovenous graft | Gy30.00 |
| Occlusion of dialysis arteriovenous fistula | Gy31.00 |
| Infection of dialysis vascular access | Gy4..00 |
| Infection of dialysis arteriovenous graft | Gy40.00 |
| Infection of dialysis arteriovenous fistula | Gy41.00 |
| Haemorrhage of dialysis vascular access | Gy5..00 |
| Haemorrhage of dialysis arteriovenous fistula | Gy51.00 |
| Rupture of dialysis arteriovenous graft | Gy60.00 |
| Nephrotic syndrome | K01..00 |
| Nephrotic syndrome with proliferative glomerulonephritis | K010.00 |
| Nephrotic syndrome with membranous glomerulonephritis | K011.00 |
| Nephrotic syndrome+membranoproliferative glomerulonephritis | K012.00 |
| Nephrotic syndrome with minimal change glomerulonephritis | K013.00 |
| Lipoid nephrosis | K013.11 |
| Steroid sensitive nephrotic syndrome | K013.12 |
| Nephrotic syndrome minor glomerular abnormality | K014.00 |
| Nephrotic syndrome focal and segmental glomerular lesions | K015.00 |
| Nephrotic syndrome diffuse membranous glomerulonephritis | K016.00 |
| Nephrotic syn difus mesangial prolifertiv glomerulonephritis | K017.00 |
| Nephrotic syn difus endocapilary proliftv glomerulonephritis | K018.00 |
| Nephrotic syn diffuse mesangiocapillary glomerulonephritis | K019.00 |
| Nephrotic syndrome dense deposit disease | K01A.00 |
| Nephrotic syndrome diffuse crescentic glomerulonephritis | K01B.00 |
| Congenital nephrotic syndrome | K01w.00 |
| Finnish nephrosis syndrome | K01w000 |
| Drash syndrome | K01w100 |
| Nephrotic syndrome with pseudohermaphroditism | K01w111 |
| Wilms' tumour + nephrotic syndrome + pseudohermaphroditism | K01w112 |
| Congenital nephrotic syndrome with focal glomerulosclerosis | K01w200 |
| Congenital nephrotic syndrome NOS | K01wz00 |
| Nephrotic syndrome in diseases EC | K01x.00 |
| Nephrotic syndrome in amyloidosis | K01x000 |
| Nephrotic syndrome in diabetes mellitus | K01x100 |
| Nephrotic syndrome in malaria | K01x200 |
| Nephrotic syndrome in polyarteritis nodosa | K01x300 |
| Nephrotic syndrome in systemic lupus erythematosus | K01x400 |
| Nephrotic syndrome with other pathological kidney lesions | K01y.00 |
| Nephrotic syndrome NOS | K01z.00 |
| Chronic rapidly progressive glomerulonephritis | K023.00 |
| Rapidly progressive nephritis unspecified | K033.00 |
| Chronic renal failure | K05..00 |
| End stage renal failure | K05..12 |
| End stage renal failure | K050.00 |
| Chronic kidney disease stage 1 | K051.00 |
| Chronic kidney disease stage 2 | K052.00 |
| Chronic kidney disease stage 3 | K053.00 |
| Chronic kidney disease stage 4 | K054.00 |
| Chronic kidney disease stage 5 | K055.00 |
| Acute nephrotic syndrm diffuse crescentic glomerulonephritis | K0A0700 |
| Rapidly progressive nephritic syndrome | K0A1.00 |
| Rapid progres nephritic syn focal+segmental glomerulr lesion | K0A1100 |
| Rapid progres neph syn diffuse membranous glomerulonephritis | K0A1200 |
| Rpd prog neph syn df mesangial prolifratv glomerulonephritis | K0A1300 |
| Rapid progres neph syn df endocapilary prolifv glomnephritis | K0A1400 |
| Rapid progressive nephritic syndrome dense deposit disease | K0A1600 |
| Rapid progres nephritic syn df crescentic glomerulonephritis | K0A1700 |
| Rapidly progressive glomerulonephritis | K0A8.00 |
| Renal tubulo-interstitial disordrs in transplant rejectn | K0B5.00 |
| End-stage renal disease | K0D..00 |
| Acute-on-chronic renal failure | K0E..00 |
| [X]Renal tubulo-interstitial disorders/transplant rejection | Kyu1C00 |
| [X]Other chronic renal failure | Kyu2100 |
| Det.ren.func.after ren.transpl | SP08011 |
| Kidney transplant failure and rejection | SP08300 |
| Acute-on-chronic rejection of renal transplant | SP08D00 |
| Acute rejection of renal transplant - grade I | SP08E00 |
| Acute rejection of renal transplant - grade II | SP08F00 |
| Acute rejection of renal transplant - grade III | SP08G00 |
| Acute rejection of renal transplant | SP08H00 |
| Chronic rejection of renal transplant | SP08J00 |
| Unexplained episode of renal transplant dysfunction | SP08N00 |
| Stenosis of vein of transplanted kidney | SP08P00 |
| Renal transplant rejection | SP08R00 |
| Urological complication of renal transplant | SP08T00 |
| Very mild acute rejection of renal transplant | SP08V00 |
| Vascular complication of renal transplant | SP08W00 |
| Disorders associated with peritoneal dialysis | SP0E.00 |
| Thrombus in peritoneal dialysis catheter | SP0E100 |
| Haemodialysis first use syndrome | SP0F.00 |
| Anaphylactoid reaction due to haemodialysis | SP0G.00 |
| Disorder associated with dialysis | SP0H.00 |
| Accid cut puncture perf h'ge - kidney dialysis | TA02000 |
| Accid cut puncture perf h'ge - perfusion NOS | TA02z00 |
| Failure of sterile precautions during perfusion | TA22.00 |
| Failure of sterile precautions during kidney dialysis | TA22000 |
| Mechanical failure of apparatus during kidney dialysis | TA42000 |
| Kidney transplant with complication, without blame | TB00100 |
| Renal transplant with complication, without blame | TB00111 |
| Kidney dialysis with complication without blame | TB11.00 |
| Renal dialysis with complication without blame | TB11.11 |
| [X]Failure sterile precautions dur kidney dialys/other perf | U612200 |
| Washing back through haemodialysis lines | Z919200 |
| [V]Kidney transplanted | ZV42000 |
| [V]Renal dialysis status | ZV45100 |
| [V]Aftercare involving intermittent dialysis | ZV56.00 |
| [V]Aftercare involving renal dialysis NOS | ZV56011 |
| [X]Other dialysis | ZVu3G00 |
| **Neurological** (epilepsy) | |
| Epilepsy | F25..00 |
| Grand mal (major) epilepsy | F251000 |
| Epileptic absences | F250011 |
| Petit mal (minor) epilepsy | F250000 |
| Temporal lobe epilepsy | F254000 |
| Fit (in known epileptic) NOS | F25z.11 |
| Status epilepticus | F253.11 |
| Traumatic epilepsy | SC20000 |
| Nocturnal epilepsy | 667B.00 |
| Epileptic seizures - myoclonic | F251300 |
| Grand mal status | F253.00 |
| Epileptic seizures - tonic | F251400 |
| Focal epilepsy | F255011 |
| Grand mal seizure | F251600 |
| Status epilepticus, unspecified | F25X.00 |
| Tonic-clonic epilepsy | F251500 |
| Jacksonian, focal or motor epilepsy | F255000 |
| Epilepsy NOS | F25z.00 |
| Petit mal status | F252.00 |
| Locl-rlt(foc)(part)idiop epilep&epilptic syn seiz locl onset | F25y200 |
| Other forms of epilepsy NOS | F25yz00 |
| Generalised nonconvulsive epilepsy | F250.00 |
| Complex partial epileptic seizure | F254500 |
| Juvenile absence epilepsy | F250400 |
| Epileptic seizures - clonic | F251200 |
| Benign Rolandic epilepsy | F25y400 |
| Juvenile myoclonic epilepsy | F25A.00 |
| Epilepsy confirmed | 1O30.00 |
| Tonic-clonic epilepsy | F251011 |
| Psychomotor epilepsy | F254100 |
| Epileptic seizures - atonic | F250200 |
| Complex partial status epilepticus | F25y300 |
| Partial epilepsy without impairment of consciousness | F255.00 |
| Generalised convulsive epilepsy | F251.00 |
| Partial epilepsy without impairment of consciousness OS | F255y00 |
| Partial epilepsy without impairment of consciousness NOS | F255z00 |
| Alcohol-induced epilepsy | F25B.00 |
| Photosensitive epilepsy | F25F.00 |
| Drug-induced epilepsy | F25C.00 |
| Epileptic seizures - akinetic | F250300 |
| Partial epilepsy with impairment of consciousness NOS | F254z00 |
| Partial epilepsy with impairment of consciousness | F254.00 |
| Epileptic automatism | F254400 |
| Psychosensory epilepsy | F254200 |
| Somatosensory epilepsy | F255200 |
| Neonatal myoclonic epilepsy | F251100 |
| Other forms of epilepsy | F25y.00 |
| Simple partial epileptic seizure | F255600 |
| Generalised convulsive epilepsy NOS | F251z00 |
| Generalised nonconvulsive epilepsy NOS | F250z00 |
| Other specified generalised convulsive epilepsy | F251y00 |
| Sensory induced epilepsy | F255100 |
| Gelastic epilepsy | F25y100 |
| Cursive (running) epilepsy | F25y000 |
| Limbic system epilepsy | F254300 |
| Visual reflex epilepsy | F255400 |
| Menstrual epilepsy | F25D.00 |
| Other specified generalised nonconvulsive epilepsy | F250y00 |
| Stress-induced epilepsy | F25E.00 |
| Motor epilepsy | F255012 |
| Unilateral epilepsy | F255500 |
| Kojevnikov's epilepsy | F257.00 |
| Visceral reflex epilepsy | F255300 |
| Partial epilepsy with autonomic symptoms | F255311 |
| Pykno-epilepsy | F250100 |
| **Neurological** (cognitive impairment) | |
| [d] senile confusion | r009.11 |
| [d]aphasia | r043.00 |
| [d]confusion | r009.00 |
| [d]dysphasia | r045100 |
| [d]memory deficit | r00z011 |
| [d]toxic confusional state | r009000 |
| [x]developmental wernicke's aphasia | eu80213 |
| [x]mild cognitive disorder | eu05700 |
| [x]psychogenic confusion | eu44y13 |
| [x]symptoms/signs inv cognit, percept, emotion state & behaviour | ryu5.00 |
| ability to process information | z7c4.00 |
| bad memory | z7ceh13 |
| cannot remember own age | z7cfs11 |
| cannot remember wedding anniversary | z7cfi00 |
| chronic confusional state | e042.00 |
| cognitive decline | 28e..00 |
| cognitive impairment | 28e3.00 |
| confusion | 2841.11 |
| delayed verbal memory | z7cgp00 |
| difficulty processing information | z7c4300 |
| difficulty processing information accurately | z7c4700 |
| difficulty processing information at normal speed | z7c4a00 |
| distortion of memory | z7cem00 |
| forgets recent activities | z7cfh00 |
| forgets what has just done | z7cfi00 |
| forgets what has just heard | z7cfm00 |
| forgets what has just read | z7cfk00 |
| forgets what has just said | z7cfj00 |
| forgets what has just seen | z7cfl00 |
| forgets what was going to do | z7cff00 |
| forgets what was going to say | z7cfg00 |
| gds level 2 - very mild cognitive decline | 3ae1.00 |
| gds level 3 - mild cognitive decline | 3ae2.00 |
| gds level 4 - moderate cognitive decline | 3ae3.00 |
| gds level 5 - moderately severe cognitive decline | 3ae4.00 |
| gds level 6 - severe cognitive decline | 3ae5.00 |
| gds level 7 - very severe cognitive decline | 3ae6.00 |
| impaired cognition | z7c1.00 |
| impairment of immediate recall | z7cea12 |
| impairment of primary memory | z7cea13 |
| impairment of working memory | z7cea11 |
| invents experiences to compensate for loss of memory | z7cen11 |
| language-related cognitive disorder | zs3..00 |
| lom - loss of memory | z7ce616 |
| long-term memory loss | z7cfo11 |
| loss of memory | z7ce615 |
| loss of memory | z7ce415 |
| loss of memory for recent events | z7cec11 |
| memory deficit | z7ceh12 |
| memory disturbance | 1b1a.13 |
| memory disturbance | z7ce414 |
| memory disturbance (& amnesia (& symptom)) | z7ce400 |
| memory dysfunction | z7ceh11 |
| memory gone | z7ce612 |
| memory impairment | z7ceh00 |
| memory impairment | 1s23.00 |
| memory lapses | z7cej00 |
| memory loss | z7ce611 |
| memory loss - amnesia | z7ce614 |
| memory loss - amnesia | 1b1a.00 |
| memory loss - amnesia | z7ce413 |
| memory loss symptom | 1b1a.12 |
| memory loss symptom | z7ce412 |
| memory problem | z7ceh14 |
| memory: import.person not knwn | 3a80.00 |
| memory: important event not kn | 3a70.00 |
| memory: own age not known | 3a10.00 |
| memory: own dob not known | 3a50.00 |
| memory: present month not knwn | 3a60.00 |
| memory: present place not knwn | 3a30.00 |
| memory: present time not known | 3a20.00 |
| memory: present year not known | 3a40.00 |
| mild cognitive impairment | 28e0.00 |
| mild memory disturbance | e2a1000 |
| mild memory disturbance | z7cel00 |
| minor memory lapses | z7cek00 |
| moderate cognitive impairment | 28e1.00 |
| no memory for recent events | z7cec12 |
| organic memory impairment | e2a1100 |
| poor long-term memory | z7cfo00 |
| poor memory | z7ceh15 |
| poor visual sequential memory | 1b1y.00 |
| reactive confusion | e132.00 |
| severe cognitive impairment | 28e2.00 |
| toxic confusional state | e030.12 |
| transient memory loss | z7ceg00 |
| unable to process information | z7c4200 |
| unable to process information accurately | z7c4600 |
| unable to process information at normal speed | z7c4900 |
| unable to recognise faces | z7c2p00 |
| unable to recognise faces by sight | z7c2r00 |
| unable to recognise familiar people | z7c2t00 |
| unable to recognise objects | z7c2h00 |
| unable to recognise objects by sight | z7c2l00 |
| unable to recognise objects visually | z7c2l11 |
| unable to recognise surroundings | z7c2600 |
| unable to remember current year | z7cfa00 |
| unable to remember day of the week | z7cfu00 |
| unable to remember motor skills | z7cfq00 |
| unable to remember new motor skills | z7cfs00 |
| unable to remember own age | z7cfs00 |
| unable to remember own date of birth | z7cfq00 |
| unable to remember today's date | z7cfw00 |
| wernicke's aphasia | zs78d13 |
| wernicke's dysphasia | zs78d00 |
| wernicke's encephalopathy | c251.11 |
| wernicke's encephalopathy | c253.00 |
| wernicke-korsakov syndrome | e011200 |
| **Cardiac** (ischaemic heart disease) | |
| ischaemic heart disease | G3...00 |
| acute myocardial infarction | G30..00 |
| transluminal balloon angioplasty of coronary artery nos | 7928z00 |
| percutaneous transluminal angioplasty of artery nec | 7A54000 |
| coronary artery bypass graft operations | 792..11 |
| coronary arteriograph.abnormal | 5543 |
| heart attack | G30..14 |
| coronary artery disease | G340.12 |
| angina on effort | G33z300 |
| angina pectoris | G33..00 |
| unstable angina | G311.13 |
| heart disease nos | G5z..00 |
| Heart disease monitoring | 662..11 |
| triple vessel disease of the heart | G340.11 |
| ischaemic heart disease nos | G3z..00 |
| mi - acute myocardial infarction | G30..15 |
| inferior myocardial infarction nos | G308.00 |
| ihd - ischaemic heart disease | G3...13 |
| other heart disease nos | G5yz.00 |
| Ventricular cardiac aneurysm | G341000 |
| coronary thrombosis | G30..12 |
| transluminal balloon angioplasty of coronary artery | 7928 |
| other specified other bypass of coronary artery | 792Dy00 |
| Cardiac disease monitoring | 662..00 |
| acute subendocardial infarction | G307.00 |
| single coronary vessel disease | G340000 |
| old myocardial infarction | G32..00 |
| crescendo angina | G311.11 |
| Ventricular fibrillation | G574000 |
| [v]presence of coronary artery bypass graft | ZV45K00 |
| cardiac enzymes abnormal | 44H3.00 |
| double coronary vessel disease | G340100 |
| other specified anterior myocardial infarction | G301.00 |
| coronary atherosclerosis | G340.00 |
| [v]presence of coronary artery bypass graft - cabg | ZV45K11 |
| percutaneous balloon coronary angioplasty | 7928.11 |
| open angioplasty of coronary artery | 7927500 |
| Aneurysm of heart | G341.00 |
| h/o: angina pectoris | 14A5.00 |
| [v]status following coronary angioplasty nos | ZV45L00 |
| other autograft bypass of coronary artery | 7921.11 |
| saphenous vein graft replacement of coronary artery os | 7920y00 |
| Ischaemic cardiomyopathy | G343.00 |
| unstable angina | G311100 |
| saphenous vein graft replacement of three coronary arteries | 7920200 |
| other autograft replacement of coronary artery nos | 7921z00 |
| saphenous vein graft replacement of two coronary arteries | 7920100 |
| syncope anginosa | G33z200 |
| ecg: myocardial infarction | 323..00 |
| ecg: myocardial ischaemia | 322..00 |
| saphenous vein graft bypass of coronary artery | 7920.11 |
| Cardiac rehabilitation | 8F9..00 |
| saphenous vein graft replacement of one coronary artery | 7920000 |
| acute inferolateral infarction | G302.00 |
| insertion of coronary artery stent | 7929400 |
| acute coronary insufficiency | G31y000 |
| other acute and subacute ischaemic heart disease | G31y.00 |
| other autograft replacement of coronary artery | 7921 |
| acute non-q wave infarction | G307000 |
| post infarct angina | G33z500 |
| Heart diseases | G....13 |
| Referral to cardiac rehabilitation nurse | 8H7v.00 |
| autograft replacement of three coronary arteries nec | 7921200 |
| coronary heart disease review | 6A4..00 |
| acute non-st segment elevation myocardial infarction | G307100 |
| variant angina pectoris | G331.11 |
| saphenous vein graft replacement of four+ coronary arteries | 7920300 |
| coronary heart disease medication review | 8B3k.00 |
| acute coronary syndrome | G311500 |
| acute anterolateral infarction | G300.00 |
| acute st segment elevation myocardial infarction | G30X000 |
| coronary artery bypass graft occlusion | SP07600 |
| stable angina | G33z700 |
| angina control | 662K.00 |
| CHD monitoring | 662N.00 |
| Cardiac diseases | G....12 |
| attack - heart | G30..11 |
| thrombosis - coronary | G30..16 |
| acute myocardial infarction nos | G30z.00 |
| angina control - improving | 662K200 |
| anterior myocardial infarction nos | G301z00 |
| lateral myocardial infarction nos | G305.00 |
| angina control nos | 662Kz00 |
| angina control - poor | 662K100 |
| dressler's syndrome | G310.11 |
| other chronic ischaemic heart disease nos | G34z.00 |
| healed myocardial infarction | G32..11 |
| mural thrombosis | G30A.00 |
| angina at rest | G311200 |
| personal history of myocardial infarction | G32..12 |
| Cardiac disease monitoring NOS | 662Z.00 |
| silent myocardial infarction | G30..17 |
| acute anteroseptal infarction | G301100 |
| percut translum thrombolysis femoral graft streptokinase | 7A4B800 |
| worsening angina | G311400 |
| nocturnal angina | G330000 |
| Chest pain on exertion | 182A.00 |
| coronary heart disease annual review | 6A2..00 |
| Coronary heart disease monitoring administration | 9Ob..00 |
| Cardiac rehabilitation class | Z677.00 |
| saphenous vein graft replacement of coronary artery | 7920 |
| [v]presence of coronary angioplasty implant and graft | ZV45800 |
| percut transluminal balloon angioplasty one coronary artery | 7928000 |
| subsequent myocardial infarction | G35..00 |
| asymptomatic coronary heart disease | G34z000 |
| [v]presence of aortocoronary bypass graft | ZV45700 |
| rotary blade coronary angioplasty | 7929300 |
| Under care of cardiac rehabilitation nurse | ZL22200 |
| Cardiovascular disease monitoring | 66f..00 |
| prosthetic replacement of coronary artery nos | 7923z00 |
| Cardiac rehabilitation declined | 8I3a.00 |
| prosthetic replacement of coronary artery | 7923 |
| autograft replacement of two coronary arteries nec | 7921100 |
| angina control - good | 662K000 |
| angina at rest | G311.14 |
| Coronary heart disease monitoring refused | 8I37.00 |
| Positive exercise ECG test | 3213111 |
| angina decubitus | G330.00 |
| atherosclerotic heart disease | G3...12 |
| peroperative angioplasty | 7A6G100 |
| transient myocardial ischaemia | G31y300 |
| endarterectomy of coronary artery nec | 792B000 |
| other specified ischaemic heart disease | G3y..00 |
| lima single anastomosis | 7925311 |
| percutaneous transluminal laser coronary angioplasty | 7929000 |
| chronic myocardial ischaemia | G34y100 |
| [V]Cardiac rehabilitation | ZV57900 |
| postmyocardial infarction syndrome | G310.00 |
| atrial septal defect/curr comp folow acut myocardal infarct | G361.00 |
| posterior myocardial infarction nos | G304.00 |
| haemopericardium/current comp folow acut myocard infarct | G360.00 |
| chronic coronary insufficiency | G34y000 |
| arteriosclerotic heart disease | G3...11 |
| Coronary heart disease monitoring 1st letter | 9Ob3.00 |
| angina pectoris nos | G33z.00 |
| new onset angina | G33z600 |
| ecg:posterior/inferior infarct | 3234 |
| ecg:shows myocardial ischaemia | 3222 |
| ecg: antero-septal infarct. | 3233 |
| Cardiac aneurysm | G341.11 |
| other acute and subacute ischaemic heart disease | G31..00 |
| other acute and subacute ischaemic heart disease nos | G31yz00 |
| other chronic ischaemic heart disease | G34..00 |
| angina pectoris nos | G33zz00 |
| acute atrial infarction | G30y000 |
| creation of bypass from mammary artery to coronary artery | 7925.11 |
| angina control - worsening | 662K300 |
| silent myocardial ischaemia | G344.00 |
| thrombosis atrium,auric append&vent/curr comp foll acute mi | G366.00 |
| acute inferoposterior infarction | G303.00 |
| acute transmural myocardial infarction of unspecif site | G30X.00 |
| angina decubitus nos | G330z00 |
| other forms of heart disease | G5...00 |
| acute q-wave infarct | G309.00 |
| cardiac rupture following myocardial infarction (mi) | G30..13 |
| suspected ischaemic heart disease | 1J61.00 |
| double implant of mammary arteries into coronary arteries | 7925100 |
| revision of bypass for three coronary arteries | 7924200 |
| allograft replacement of coronary artery | 7922 |
| postoperative myocardial infarction | G38..00 |
| ischaemic chest pain | G33z400 |
| h/o: heart disease nos | 14AA.00 |
| allograft bypass of coronary artery | 7922.11 |
| acute posterolateral myocardial infarction | G30B.00 |
| revision of bypass for coronary artery | 7924 |
| other bypass of coronary artery nos | 792Dz00 |
| percut transluminal coronary thrombolysis with streptokinase | 7929100 |
| double anastomosis of mammary arteries to coronary arteries | 7925000 |
| percut translum balloon angioplasty mult coronary arteries | 7928100 |
| Coronary heart disease monitoring 2nd letter | 9Ob4.00 |
| refractory angina | G311300 |
| Coronary heart disease monitoring 3rd letter | 9Ob5.00 |
| other specified chronic ischaemic heart disease | G34y.00 |
| other acute myocardial infarction | G30y.00 |
| ecg: q wave | 32B..00 |
| other bypass of coronary artery | 792D.00 |
| post infarction pericarditis | G501.00 |
| Refuses coronary heart disease monitoring | 9Ob1.00 |
| ecg: myocardial ischaemia nos | 322Z.00 |
| Attends coronary heart disease monitoring | 9Ob0.00 |
| angiocardiography abnormal | 5533 |
| h/o: myocardial infarct <60 | 14A3.00 |
| other specified chronic ischaemic heart disease nos | G34yz00 |
| prosthetic bypass of coronary artery | 7923.11 |
| other specified heart disease | G5y..00 |
| certain current complication follow acute myocardial infarct | G36..00 |
| preinfarction syndrome | G311.00 |
| atherosclerotic cardiovascular disease | G342.00 |
| coronary artery spasm | G332.00 |
| ventric septal defect/curr comp fol acut myocardal infarctn | G362.00 |
| connection of mammary artery to coronary artery | 7925 |
| connection of mammary artery to coronary artery os | 7925y00 |
| Coronary heart disease monitoring verbal invitation | 9Ob6.00 |
| Cardiac rehabilitation - phase 1 | 8F90.00 |
| Cardiac rehabilitation - phase 2 | 8F91.00 |
| subsequent myocardial infarction of inferior wall | G351.00 |
| rotary blade angioplasty | 7A54500 |
| coronary thrombosis not resulting in myocardial infarction | G312.00 |
| Coronary heart disease monitoring check done | 9Ob8.00 |
| [x]other forms of angina pectoris | Gyu3000 |
| impending infarction | G311.12 |
| subendocardial ischaemia | G31y200 |
| ecg: old myocardial infarction | 3232 |
| h/o: myocardial infarct >60 | 14A4.00 |
| acute anteroapical infarction | G301000 |
| percut translum coronary thrombolytic therapy- streptokinase | 7929111 |
| Cardiac rehabilitation - phase 3 | 8F92.00 |
| Other ill-defined heart disease NOS | G5yyz00 |
| acute septal infarction | G30y200 |
| transluminal balloon angioplasty of coronary artery os | 7928y00 |
| Aneurysm of heart NOS | G341z00 |
| postoperative subendocardial myocardial infarction | G384.00 |
| ecg: s-t depression | 32E4.00 |
| insertion of drug-eluting coronary artery stent | 7929500 |
| percut translum balloon angioplasty bypass graft coronary a | 7928200 |
| autograft replacement of four of more coronary arteries nec | 7921300 |
| perc translumin balloon angioplasty stenting coronary artery | 793G.00 |
| Plasma troponin T level | 44MH.00 |
| percutaneous transluminal thrombolysis of artery | 7A54700 |
| autograft replacement of one coronary artery nec | 7921000 |
| allograft replacement of four or more coronary arteries | 7922300 |
| h/o: treatment for ischaemic heart disease | 14AL.00 |
| subsequent myocardial infarction of anterior wall | G350.00 |
| allograft replacement of three coronary arteries | 7922200 |
| antianginal therapy | 8B27.00 |
| other acute myocardial infarction nos | G30yz00 |
| postoperative transmural myocardial infarction anterior wall | G380.00 |
| subsequent myocardial infarction of unspecified site | G35X.00 |
| ecg: q wave abnormal | 32B2.00 |
| postoperative transmural myocardial infarction inferior wall | G381.00 |
| Cardiac rehabilitation - phase 4 | 8F93.00 |
| [x]other forms of chronic ischaemic heart disease | Gyu3300 |
| Coronary heart disease monitoring default | 9Ob2.00 |
| allograft replacement of coronary artery nos | 7922z00 |
| Cardiovascular disease interim monitoring | 66f1.00 |
| Plasma creatinine phosphokinase MB isoenzyme level | 44HJ.00 |
| h/o: myocardial infarction in last year | 14AH.00 |
| single anastomosis of mammary artery to coronary artery nec | 7925300 |
| saphenous vein graft replacement coronary artery nos | 7920z00 |
| [x]ischaemic heart diseases | Gyu3.00 |
| ecg: lateral infarction | 3236 |
| revision of bypass for one coronary artery | 7924000 |
| preinfarction syndrome nos | G311z00 |
| stenocardia | G33z100 |
| replacement of coronary arteries using multiple methods | 792C000 |
| mi - myocardial infarction aborted | G311011 |
| ecg: subendocardial infarct | 3235 |
| other replacement of coronary artery | 792C.00 |
| connection of mammary artery to coronary artery nos | 7925z00 |
| h/o: angina in last year | 14AJ.00 |
| allograft replacement of two coronary arteries | 7922100 |
| revision of bypass for coronary artery nos | 7924z00 |
| radionuclide heart study abnormal | 5C11.00 |
| ecg: myocardial infarct nos | 323Z.00 |
| ruptur cardiac wall w'out haemopericard/cur comp fol ac mi | G363.00 |
| other specified allograft replacement of coronary artery | 7922y00 |
| percutaneous transluminal arterial thrombolysis reconstruct | 7A56000 |
| ruptur chordae tendinae/curr comp fol acute myocard infarct | G364.00 |
| perc translum ball angio insert 1-2 drug elut stents cor art | 793G000 |
| cardiac enzymes abnormal - first set | 44H3000 |
| single implantation thoracic artery into coronary artery nec | 7926300 |
| myocardial infarction aborted | G311000 |
| perc translum balloon angioplasty stenting coronary art nos | 793Gz00 |
| other autograft replacement of coronary artery os | 7921y00 |
| diab mellit insulin-glucose infus acute myocardial infarct | 889A.00 |
| ecg: q wave pathological | 32B3.00 |
| double anastom thoracic arteries to coronary arteries nec | 7926000 |
| acute papillary muscle infarction | G30y100 |
| true posterior myocardial infarction | G306.00 |
| prosthetic graft patch angioplasty | 7A6H300 |
| prosthetic replacement of three coronary arteries | 7923200 |
| ecg: q wave nos | 32BZ.00 |
| status anginosus | G33z000 |
| prosthetic replacement of two coronary arteries | 7923100 |
| percutaneous transluminal angioplasty of vascular graft | 7A6H400 |
| Other cardiac wall aneurysm | G341100 |
| revision of bypass for two coronary arteries | 7924100 |
| single anastomosis of thoracic artery to coronary artery nec | 7926200 |
| prosthetic replacement of four or more coronary arteries | 7923300 |
| rima single anastomosis | 7925312 |
| single implantation of mammary artery into coronary artery | 7925400 |
| microinfarction of heart | G31y100 |
| [x]other forms of acute ischaemic heart disease | Gyu3200 |
| postoperative myocardial infarction, unspecified | G38z.00 |
| [x]other forms of heart disease | Gyu5.00 |
| rupture papillary muscle/curr comp fol acute myocard infarct | G365.00 |
| mechanical complication of coronary bypass | SP00300 |
| allograft replacement of one coronary artery | 7922000 |
| Coronary heart disease monitoring telephone invite | 9Ob9.00 |
| percutaneous transluminal atherectomy | 7A54800 |
| replacement of coronary artery nos | 792Cz00 |
| subsequent myocardial infarction of other sites | G353.00 |
| connection of other thoracic artery to coronary artery nos | 7926z00 |
| percutaneous transluminal venous thrombolysis nec | 7A6S300 |
| perc translum balloon angioplasty insert 1-2 stents cor art | 793G200 |
| percut translum cutting balloon angioplasty coronary artery | 7928300 |
| percutaneous transluminal balloon angioplasty of artery | 7A56400 |
| perc tran ball angio ins 3 or more drug elut stents cor art | 793G100 |
| prosthetic replacement of one coronary artery | 7923000 |
| percutaneous cor balloon angiop 3 more stents cor art nec | 793G300 |
| percutaneous transluminal atherectomy of coronary artery | 7929600 |
| other specified replacement of coronary artery | 792Cy00 |
| admit ischaemic heart disease emergency | 8H2V.00 |
| os perc translumina balloon angioplast stenting coronary art | 793Gy00 |
| connection of other thoracic artery to coronary artery | 7926 |
| [x]acute transmural myocardial infarction of unspecif site | Gyu3400 |
| cardiac troponin positive | 44p2.00 |
| other specified revision of bypass for coronary artery | 7924y00 |
| [x]subsequent myocardial infarction of unspecified site | Gyu3600 |
| history of myocardial infarction | 14AT.00 |
| [x]atherosclerosis of other arteries | Gyu7000 |
| coronary artery bypass graft operation planned | 8L40.00 |
| coronary angiography planned | 8LF..00 |
| coronary angioplasty planned | 8L41.00 |
| long term dual antiplatelet drug therapy indicated | 8BGC.00 |
| frequency of angina | 187..00 |
| coronary heart disease care plan | 8CMP.00 |
| h/o acute coronary syndrome | 14AW.00 |
| coronary microvascular disease | G39..00 |
| postoperative transmural myocardial infarction unspec site | G383.00 |
| referral to angina plan self-management programme | 8T04.00 |
| angina self-management plan agreed | 661M000 |
| referral to angina plan self-management programme declined | 8IEY.00 |
| **Cardiac** (hypertension) | |
| hypertensive disease | G2...00 |
| high blood pressure | G20..11 |
| essential hypertension | G20..00 |
| benign essential hypertension | G201.00 |
| h/o: hypertension | 14A2.00 |
| hypertension resolved | 2126100 |
| on treatment for hypertension | 662O.00 |
| hypertension nos | G20z.11 |
| hypertensive encephalopathy | G672.00 |
| seen in hypertension clinic | 9N03.00 |
| systolic hypertension | G202.00 |
| hypertension monitoring | 662..12 |
| hypertensive renal disease | G22..00 |
| hypertension monitoring admin. | 9OI..00 |
| referral to hypertension clinic | 8HT5.00 |
| hypertensive retinopathy | F421300 |
| hypertensive disease nos | G2z..00 |
| secondary hypertension | G24..00 |
| borderline hyperten:yearly obs | 6624 |
| bp - hypertensive disease | G2...11 |
| cardiomegaly - hypertensive | G21z011 |
| white coat hypertension | 246M.00 |
| essential hypertension nos | G20z.00 |
| excepted from hypertension qual indicators: patient unsuit | 9h31.00 |
| excepted from hypertension qual indicators: informed dissent | 9h32.00 |
| patient on maximal tolerated antihypertensive therapy | 8BL0.00 |
| hypertension clinical management plan | 8CR4.00 |
| hypertension treatm.stopped | 662H.00 |
| hypertension monitoring | 662P.00 |
| hypertensive treatm.changed | 662G.00 |
| hypertensive renal disease nos | G22z.00 |
| malignant essential hypertension | G200.00 |
| secondary hypertension nos | G24z.00 |
| hypertensive heart disease nos | G21zz00 |
| hypertensive heart disease | G21..00 |
| good hypertension control | 6627 |
| nephrosclerosis | G22..11 |
| antihypertensive therapy | 8B26.00 |
| hypertension six month review | 662c.00 |
| moderate hypertension control | 662b.00 |
| other specified hypertensive disease | G2y..00 |
| hypertension annual review | 662d.00 |
| hypertension resolved | 212K.00 |
| adverse reaction to antihypertensives nos | TJC7z00 |
| adverse reaction to other antihypertensives | TJC7.00 |
| hypertension treatm. started | 662F.00 |
| hypertensive heart&renal dis wth (congestive) heart failure | G232.00 |
| hypertension treatment refused | 8I3N.00 |
| suspected hypertension | 1JD..00 |
| hypertension monitored | 9OIA.11 |
| secondary benign renovascular hypertension | G241000 |
| poor hypertension control | 6628 |
| hypertension clinic admin. | 9OI..11 |
| seen in hypertension clinic | 9N1y200 |
| hypertensive heart and renal disease with renal failure | G233.00 |
| hyperten.monitor offer default | 9OI3.00 |
| hypertens.monitor phone invite | 9OI8.00 |
| renal hypertension | G22z.11 |
| [x] adverse reaction to other antihypertensives | U60C511 |
| hypertension:follow-up default | 6629 |
| hypertens.monitor.1st letter | 9OI4.00 |
| hypertens.monitor 2nd letter | 9OI5.00 |
| hypertens.monitor 3rd letter | 9OI6.00 |
| hypertension secondary to drug | G24z100 |
| secondary renovascular hypertension nos | G24z000 |
| hypertensive heart disease nos | G21z.00 |
| secondary malignant hypertension | G240.00 |
| hypertensive crisis | G672.11 |
| hypertensive renal disease with renal failure | G222.00 |
| exception reporting: hypertension quality indicators | 9h3..00 |
| hypertens.monitoring admin.nos | 9OIZ.00 |
| dna - did not attend hypertension clinic | 9N4L.00 |
| hypertension secondary to endocrine disorders | G244.00 |
| hypertension monitor.chck done | 9OIA.00 |
| blind hypertensive eye | F404200 |
| malignant hypertensive renal disease | G220.00 |
| hypertens.monitor verbal inv. | 9OI7.00 |
| secondary hypertension nos | G24zz00 |
| refuses hypertension monitor. | 9OI2.00 |
| pre-eclampsia or eclampsia with pre-existing hypertension | L127.00 |
| benign hypertensive renal disease | G221.00 |
| [x] adverse reaction to antihypertensives nos | U60C51A |
| pre-exist hypertension compl preg childbirth and puerperium | L128.00 |
| attends hypertension monitor. | 9OI1.00 |
| malignant hypertensive heart disease | G210.00 |
| secondary benign hypertension nos | G241z00 |
| benign hypertensive heart disease with ccf | G211100 |
| benign hypertensive heart disease | G211.00 |
| pre-exist 2ndry hypertens comp preg childbth and puerperium | L128200 |
| secondary benign hypertension | G241.00 |
| hyperten heart&renal dis+both(congestv)heart and renal fail | G234.00 |
| secondary malignant renovascular hypertension | G240000 |
| pre-exist hyperten heart dis compl preg childbth+puerperium | L128000 |
| hypertensive heart disease nos without ccf | G21z000 |
| benign hypertensive heart disease without ccf | G211000 |
| other pre-existing hypertension in preg/childb/puerp nos | L122z00 |
| hypertensive heart disease nos with ccf | G21z100 |
| benign hypertensive heart and renal disease | G231.00 |
| [x]oth antihyperten drug caus advers eff in therap use, nec | U60C500 |
| hypertensive heart and renal disease | G23..00 |
| other pre-existing hypertension in preg/childbirth/puerp | L122.00 |
| hypertens.monitor deleted | 9OI9.00 |
| malignant hypertensive heart and renal disease | G230.00 |
| hypertensive heart and renal disease nos | G23z.00 |
| [x]hypertensive diseases | Gyu2.00 |
| other pre-existing hypertension in preg/childb/puerp - deliv | L122100 |
| malignant hypertensive heart disease with ccf | G210100 |
| secondary malignant hypertension nos | G240z00 |
| other pre-existing hypertension in preg/childb/puerp unspec | L122000 |
| diastolic hypertension | G203.00 |
| high cost hypertension drugs | 7Q01.00 |
| pre-eclampsia or eclampsia + pre-existing hypertension nos | L127z00 |
| malignant hypertensive heart disease without ccf | G210000 |
| trial withdrawal of antihypertensive therapy | 662r.00 |
| other pre-exist hypertension in preg/childb/puerp-not deliv | L122300 |
| [x]hypertension secondary to other renal disorders | Gyu2100 |
| lifestyle advice regarding hypertension | 67H8.00 |
| trial reduction of antihypertensive therapy | 662q.00 |
| other specified high cost hypertension drugs | 7Q01y00 |
| hypertension 9 month review | 662P000 |
| [x]other secondary hypertension | Gyu2000 |
| malignant hypertensive heart disease nos | G210z00 |
| stage 2 hypertension (nice - nat ins for hth clin excl 2011) | G28..00 |
| stage 1 hypertension | G25..11 |
| stage 1 hypertension (nice - nat ins for hth clin excl 2011) | G25..00 |
| hypertension resistant to drug therapy | G27..00 |
| severe hypertension | G26..11 |
| benign hypertensive heart disease nos | G211z00 |
| severe hypertension (nat inst for health clinical ex 2011) | G26..00 |
| primary hypertension | G20..12 |
| **Gastrointestinal** (irritable bowel syndrome) | |
| Irritable colon - Irritable bowel syndrome | J521.00 |
| Irrigation of bowel NEC | 773C400 |
| History of irritable bowel syndrome | 14CF.00 |
| Management of irritable bowel syndrome | 8Cm..00 |
| Irritable bowel syndrome with diarrhoea | J521000 |
| Irritable bowel syndrome | J521.11 |
| **Gastrointestinal** (inflammatory bowel disease) | |
| Crohn's disease | J40..11 |
| Ulcerative colitis | J410100 |
| Ulcerative colitis and/or proctitis | J41..12 |
| Inflammatory bowel disease | J4...12 |
| Idiopathic proctocolitis | J41..00 |
| H/O: ulcerative colitis | 14C4.11 |
| Crohn's colitis | J401z11 |
| Ulcerative proctocolitis | J410.00 |
| Ulcerative proctitis | J410300 |
| Crohn's disease of the small bowel NOS | J400z00 |
| CDAI - Crohn's disease activity index | ZR3S.11 |
| Regional enteritis - Crohn's disease | J40..00 |
| Crohn's disease activity index | ZR3S.00 |
| Juvenile arthritis in Crohn's disease | N045300 |
| Idiopathic proctocolitis NOS | J41z.00 |
| Regional ileocolitis | J402.00 |
| Arthropathy in ulcerative colitis | N031000 |
| Arthropathy in Crohn's disease | N031100 |
| Crohn's disease of the large bowel NOS | J401z00 |
| Exacerbation of ulcerative colitis | J410400 |
| Other idiopathic proctocolitis | J41y.00 |
| Ulcerative rectosigmoiditis | J410200 |
| Crohn's disease of the terminal ileum | J400200 |
| Orofacial Crohn's disease | J08z900 |
| Ulcerative (chronic) enterocolitis | J411.00 |
| Ulcerative proctocolitis NOS | J410z00 |
| Exacerbation of Crohn's disease of small intestine | J400500 |
| Exacerbation of Crohn's disease of large intestine | J401200 |
| Crohn's disease of the ileum NOS | J400400 |
| Ulcerative (chronic) ileocolitis | J412.00 |
| Other idiopathic proctocolitis NOS | J41yz00 |
| Regional enteritis of the large bowel | J401.00 |
| Ulcerative ileocolitis | J410000 |
| Regional enteritis of the small bowel | J400.00 |
| Granulomatous enteritis | J40..12 |
| Regional enteritis NOS | J40z.00 |
| [X]Other ulcerative colitis | Jyu4100 |
| Crohn's disease NOS | J40z.11 |
| Regional enteritis of the colon | J401000 |
| Regional enteritis of the jejunum | J400100 |
| Regional enteritis of the rectum | J401100 |
| Crohn's disease of the ileum unspecified | J400300 |
| [X]Other Crohn's disease | Jyu4000 |
| Juvenile arthritis in ulcerative colitis | N045400 |
| Regional enteritis of the duodenum | J400000 |
| **Respiratory** (acute respiratory tract infection) | |
| chest infection | H06z011 |
| acute bronchitis | H060.00 |
| influenza | H27..00 |
| pneumonia due to unspecified organism | H26..00 |
| whooping cough | A33..00 |
| bronchopneumonia due to unspecified organism | H25..00 |
| acute bronchiolitis | H061.00 |
| croup | H044.00 |
| acute tracheitis | H041.00 |
| laryngotracheitis | H042.11 |
| acute viral bronchitis unspecified | H060w00 |
| pneumonia due to mycoplasma pneumoniae | H231.00 |
| lobar (pneumococcal) pneumonia | H21..00 |
| laryngotracheobronchitis | H301.00 |
| flu like illness | H27z.11 |
| chest cold | H07..00 |
| chest infection nos | H06z000 |
| tracheobronchitis nos | H300.00 |
| lower resp tract infection | H06z100 |
| basal pneumonia due to unspecified organism | H261.00 |
| bordetella pertussis | A330.00 |
| viral pneumonia | H20..00 |
| atypical pneumonia | H28..00 |
| pneumonia due to staphylococcus | H224.00 |
| influenza like illness | H27z.12 |
| acute wheezy bronchitis | H060.11 |
| pneumonia or influenza nos | H2z..00 |
| acute lower respiratory tract infection | H062.00 |
| notification of whooping cough | 65VA.00 |
| influenza-like symptoms | 16L..00 |
| acute pneumococcal bronchitis | H060600 |
| chest infection - viral pneumonia | H20..11 |
| lobar pneumonia due to unspecified organism | H260.00 |
| tuberculous pneumonia | A116.00 |
| pneumonia and influenza | H2...00 |
| acute laryngotracheitis | H042.00 |
| tracheopharyngitis | H053.00 |
| acute purulent bronchitis | H060300 |
| acute tracheobronchitis | H060500 |
| other specified pneumonia or influenza | H2y..00 |
| pneumonia - legionella | H22y200 |
| pneumonia due to streptococcus | H223.00 |
| acute tracheitis without obstruction | H041000 |
| influenza with bronchopneumonia | H270000 |
| influenza with gastrointestinal tract involvement | H27y100 |
| viral pneumonia nos | H20z.00 |
| legionella | A3A4.00 |
| bordetella parapertussis | A331.00 |
| influenza with laryngitis | H271000 |
| influenza with pneumonia | H270.00 |
| acute laryngitis and tracheitis nos | H04z.00 |
| chest infection - unspecified bronchopneumonia | H25..11 |
| acute tracheitis nos | H041z00 |
| influenza nos | H27z.00 |
| chlamydial pneumonia | H233.00 |
| acute bronchiolitis with bronchospasm | H061200 |
| chest infection - unspecified bronchitis | H30..11 |
| acute bronchiolitis nos | H061z00 |
| acute bronchiolitis due to respiratory syncytial virus | H061500 |
| chest infection - pnemonia due to unspecified organism | H26..11 |
| croup | H043211 |
| acute bronchitis nos | H060z00 |
| chronic obstruct pulmonary dis with acute lower resp infectn | H3y0.00 |
| acute croupous bronchitis | H060400 |
| pharyngotracheitis | H052.00 |
| acute haemophilus influenzae bronchitis | H060800 |
| chest infection - other bacterial pneumonia | H22..11 |
| bronchiolitis obliterans organising pneumonia | H564.00 |
| bacterial pneumonia nos | H22z.00 |
| hypostatic pneumonia | H540000 |
| influenza with respiratory manifestations nos | H271z00 |
| pneumonia due to klebsiella pneumoniae | H220.00 |
| pneumonia with varicella | H24y700 |
| chest infection with infectious disease ec | H24..11 |
| hypostatic bronchopneumonia | H540100 |
| acute laryngotracheitis nos | H042z00 |
| acute bacterial bronchitis unspecified | H060x00 |
| acute laryngotracheitis without obstruction | H042000 |
| varicella pneumonitis | A521.00 |
| pneumonia due to other specified organisms | H23..00 |
| pneumonia with pneumocystis carinii | H24y200 |
| hiv disease resulting in pneumocystis carinii pneumonia | A789300 |
| other bacterial pneumonia | H22..00 |
| chest infection - pneumococcal pneumonia | H21..11 |
| acute bronchitis due to parainfluenza virus | H060C00 |
| chest infection - influenza with pneumonia | H270.11 |
| influenza with pharyngitis | H271100 |
| acute bronchitis and bronchiolitis | H06..00 |
| pneumonia with whooping cough | H243.00 |
| post operative chest infection | SP13200 |
| pneumonia due to pseudomonas | H221.00 |
| chest infection - pneumonia organism os | H23..11 |
| mycoplasma pneumoniae [pplo] cause/dis classifd/oth chaptr | A3BXA00 |
| pneumonia due to respiratory syncytial virus | H201.00 |
| influenza with other manifestations nos | H27yz00 |
| staphylococcal pleurisy | H510A00 |
| bacterial pleurisy with effusion | H511.00 |
| acute bronchitis due to mycoplasma pneumoniae | H060A00 |
| postmeasles pneumonia | A551.00 |
| pneumococcal pleurisy | H510900 |
| legionella antibody positive | 43D3.00 |
| viral pneumonia nec | H20y.00 |
| pneumonia due to specified organism nos | H23z.00 |
| pneumonia with aspergillosis | H246.00 |
| postoperative pneumonia | H262.00 |
| pneumonia with pertussis | H243.11 |
| abscess of lung with pneumonia | H530300 |
| pneumocystosis | AD63.00 |
| influenza with pneumonia nos | H270z00 |
| pneumonia due to parainfluenza virus | H202.00 |
| acute lower respiratory tract infection | H06z112 |
| pneumonia due to haemophilus influenzae | H222.00 |
| pneumonia - candidal | AB24.11 |
| pneumonia with infectious diseases ec | H24..00 |
| pneumonia with measles | H240.00 |
| acute bronchitis or bronchiolitis nos | H06z.00 |
| acute laryngitis and tracheitis | H04..00 |
| primary pulmonary blastomycosis | AB50100 |
| whooping cough nos | A33z.00 |
| pneumonia with cytomegalic inclusion disease | H241.00 |
| pneumococcal pleurisy with effusion | H511000 |
| acute streptococcal bronchitis | H060700 |
| influenza with other respiratory manifestation | H271.00 |
| pneumonia due to bacteria nos | H22yz00 |
| bacterial pleurisy with effusion nos | H511z00 |
| cytomegaloviral pneumonitis | A785000 |
| pneumonia due to proteus | H22y100 |
| severe acute respiratory syndrome | H20y000 |
| influenza with encephalopathy | H27y000 |
| pneumonic plague, unspecified | A205.00 |
| notification of legionella | 65VF.00 |
| influenza with other manifestations | H27y.00 |
| herpes simplex pneumonia | A54x400 |
| acute bronchitis due to respiratory syncytial virus | H060D00 |
| pneumonia due to haemophilus influenzae | H222.11 |
| pneumonia with typhoid fever | H24y600 |
| acute neisseria catarrhalis bronchitis | H060900 |
| acute fibrinous bronchitis | H060000 |
| ornithosis with pneumonia | A730.00 |
| pneumonia due to other specified bacteria | H22y.00 |
| pleuropneumonia-like organism (pplo) infection | A3By400 |
| pneumonia with candidiasis | H247000 |
| pneumonia due to other aerobic gram-negative bacteria | H22yX00 |
| [x]other viral pneumonia | Hyu0800 |
| [x]other pneumonia, organism unspecified | Hyu0H00 |
| whooping cough - other specified organism | A33y.00 |
| [x]pneumonia in viral diseases classified elsewhere | Hyu0D00 |
| pneumonia with systemic mycosis nos | H247z00 |
| acute capillary bronchiolitis | H061000 |
| primary pulmonary coccidioidomycosis | AB30.00 |
| pulmonary cryptococcosis | AB65000 |
| acute myocarditis - influenzal | G520300 |
| toxoplasma pneumonitis | AD04.00 |
| gangrenous pneumonia | H530200 |
| salmonella pneumonia | A022200 |
| pulmonary histoplasmosis | AB42.00 |
| pneumonia due to eaton's agent | H230.00 |
| e.coli pneumonia | H22y011 |
| pneumonia with q-fever | H24y300 |
| eaton's agent infection | A3By100 |
| pneumonia with actinomycosis | H24y000 |
| pneumonia with ornithosis | H242.00 |
| influenza with pneumonia, influenza virus identified | H270100 |
| avian influenza virus nucleic acid detection | 43jQ.00 |
| [x]other bacterial pneumonia | Hyu0A00 |
| pneumonia due to streptococcus, group b | H223000 |
| other whooping cough nos | A33yz00 |
| pulmonary actinomycosis | A391.00 |
| acute bronchitis due to rhinovirus | H060E00 |
| pneumonia due to escherichia coli | H22y000 |
| acute bronchitis due to echovirus | H060F00 |
| acute bronchiolitis due to other specified organisms | H061600 |
| pneumonia with infectious diseases ec nos | H24z.00 |
| [x]other acute lower respiratory infections | Hyu1.00 |
| pneumonia due to adenovirus | H200.00 |
| pneumonia with nocardiasis | H24y100 |
| acute tracheitis with obstruction | H041100 |
| acute exudative bronchiolitis | H061300 |
| streptococcal pleurisy | H510B00 |
| pneumonia with other infectious diseases ec | H24y.00 |
| acute laryngotracheitis with obstruction | H042100 |
| pneumonia with other infectious diseases ec nos | H24yz00 |
| primary pneumonic plague | A203.00 |
| acute pseudomembranous bronchitis | H060200 |
| pneumonia with salmonellosis | H24y400 |
| [x]acute bronchitis due to other specified organisms | Hyu1000 |
| pneumonia due to pleuropneumonia like organisms | H232.00 |
| parainfluenza type 3 nucleic acid detection | 43jz.00 |
| acute pulmonary histoplasmosis capsulati | AB40600 |
| staphylococcal pleurisy with effusion | H511100 |
| acute bronchitis due to coxsackievirus | H060B00 |
| parainfluenza type 1 nucleic acid detection | 43jx.00 |
| parainfluenza type 2 nucleic acid detection | 43jy.00 |
| avian influenza | H29..00 |
| influenza b virus detected | 4JU5.00 |
| influenza h3 virus detected | 4JU2.00 |
| influenza h1 virus detected | 4JU0.00 |
| mycoplasma pneumoniae detected | 4JUK.00 |
| human parainfluenza virus detected | 4JUF.00 |
| influenza a virus, other or untyped strain detected | 4JU4.00 |
| [x]influenza+other manifestations, virus not identified | Hyu0700 |
| [x]influenza+oth respiratory manifestatns,virus not identifd | Hyu0600 |
| [x]influenza+other manifestations,influenza virus identified | Hyu0500 |
| influenza a (h1n1) swine flu | H2A..11 |
| possible influenza a virus h1n1 subtype | 1W0..00 |
| suspected swine influenza | 1J72.11 |
| suspected influenza a virus subtype h1n1 infection | 1J72.00 |
| influenza due to influenza a virus subtype h1n1 | H2A..00 |
| influenza a virus h1n1 subtype detected | 4J3L.00 |
| influenza h5 virus detected | 4JU3.00 |
| [x]flu+oth respiratory manifestations,'flu virus identified | Hyu0400 |
| [x]pneumonia due to other specified infectious organisms | Hyu0B00 |
| pneumonia with toxoplasmosis | H24y500 |
| [x]acute bronchiolitis due to other specified organisms | Hyu1100 |
| [x]whooping cough, unspecified | Ayu3A00 |
| histoplasma duboisii with pneumonia | AB41500 |
| histoplasma capsulatum with pneumonia | AB40500 |
| acute membranous bronchitis | H060100 |
| sars virus nucleic acid detection | 43jn.00 |
| influenza h2 virus detected | 4JU1.00 |
| pneumonia with coccidioidomycosis | H247100 |
| cryptogenic organising pneumonia | H564.11 |
| influenza a nucleic acid detection | 43w6.00 |
| community acquired pneumonia | H2B..00 |
| hospital acquired pneumonia | H2C..00 |
| parainfluenza type 4 nucleic acid detection | 43w9.00 |
| influenza b nucleic acid detection | 43wD.00 |
| acute bronchiolitis due to human metapneumovirus | H061700 |
| [x]mycoplasma pneumoniae [pplo]cause/dis classifd/oth chaptr | AyuK900 |
| pneumonia due to human metapneumovirus | H203.00 |
| pneumonia with tularaemia | H244.00 |
| streptococcal pleurisy with effusion | H511200 |
| pneumonia with other systemic mycoses | H247.00 |
| bordetella pertussis deoxyribonucleic acid detection | 43wE.00 |
| [x]pneumonia in bacterial diseases classified elsewhere | Hyu0C00 |
| [x]pneumonia in other diseases classified elsewhere | Hyu0G00 |

**Appendix C: List of Charlson co-morbidities [Ref: 31]**

| **Co-morbidity** |
| --- |
| Acquired immunodeficiency syndrome |
| Diabetes |
| Diabetes uncomplicated |
| Rheumatic disease |
| Renal disease |
| Peripheral vascular disease |
| Peptic ulcer disease |
| Myocardial infarction |
| Moderate or severe liver disease |
| Mild liver disease |
| Metastatic solid tumour |
| Hemiplegia or paraplegia |
| Dementia |
| Congestive heart failure |
| Chronic pulmonary disease |
| Cerebrovascular disease |
| Cancer |

**Appendix D: Disease-specific incidence rates per 10,000 person-years at risk among individuals exposed to Shiga-toxin producing *Escherichia coli* O157 infection (STEC O157), Wales, 1990-2020. Outcomes were defined using clinical code lists for General Practitioner (GP) practice consultations and/or hospital admissions (“Primary and/or secondary care”)**

| **Outcome** |  | **Condition** | **Incidence (per 10,000 person-years at risk)** |
| --- | --- | --- | --- |
| Primary and/or secondary care | | | |
|  | Kidney | Chronic kidney disease stage 3-5 | 12.0 |
|  |  | End stage kidney disease | 10.7 |
|  |  | Chronic kidney disease stage 1-2 | 7.0 |
|  |  | Chronic kidney disease unspecified | 4.4 |
|  | Neurological | Cognitive impairment | 20.7 |
|  |  | Epilepsy | 5.0 |
|  | Cardiac | Hypertension | 64.4 |
|  |  | Ischaemic heart disease | 21.7 |
|  | Gastrointestinal | Irritable bowel syndrome | 45.0 |
|  |  | Inflammatory bowel disease | 24.1 |
|  | Respiratory | Infective respiratory disease | 288.4 |
|  | Endocrine | Type 2 diabetes | 19.5 |
|  |  | Type 1 diabetes or undetermined type diabetes | 6.3 |

**Appendix E: Smoothed survival curves showing time (years) to first kidney complication among individuals exposed to Shiga toxin-producing Escherichia coli O157 infection (STEC O157) (first row) or STEC O157 with haemolytic uraemic syndrome (STEC O157-HUS) (second row) at ≥1 year follow-up, January 1990-January 2020, Wales. Censoring events were defined as the first of: death, emigration, or study end (1 January 2020). Outcomes were defined using kidney clinical code lists for General Practitioner (GP) practice consultations (“Primary care only”) and/or hospital admissions (“Primary and/or secondary care”). Unexposed cohorts were individuals registered with a GP practice in Wales who were the same age and sex as their exposed equivalent at time of STEC O157 positive specimen date or symptom onset**


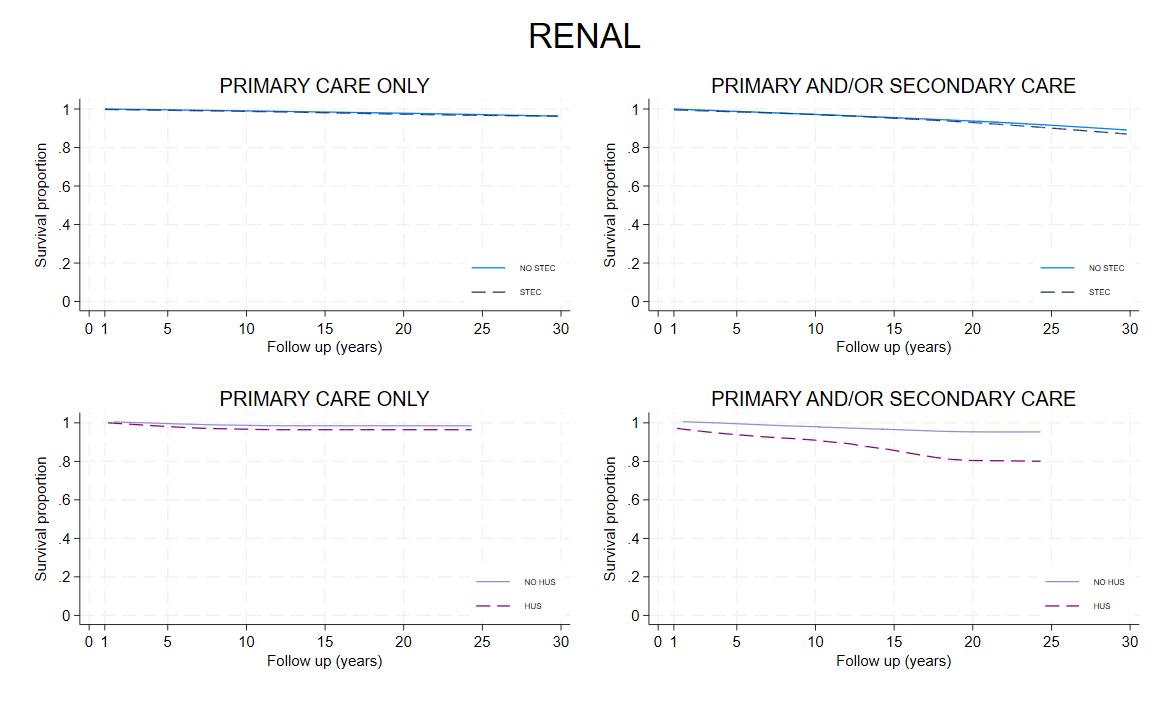


**Appendix F: Smoothed survival curves showing time (years) to first neurological complication among individuals exposed to Shiga toxin-producing Escherichia coli O157 infection (STEC O157) (first row) or STEC O157 with haemolytic uraemic syndrome (STEC O157-HUS) (second row) at ≥1 year follow-up, January 1990-January 2020, Wales. Censoring events were defined as the first of: death, emigration, or study end (1 January 2020). Outcomes were defined using neurological clinical code lists for General Practitioner (GP) practice consultations (“Primary care only”) and/or hospital admissions (“Primary and/or secondary care”). Unexposed cohorts were individuals registered with a GP practice in Wales who were the same age and sex as their exposed equivalent at time of STEC O157 positive specimen date or symptom onset**


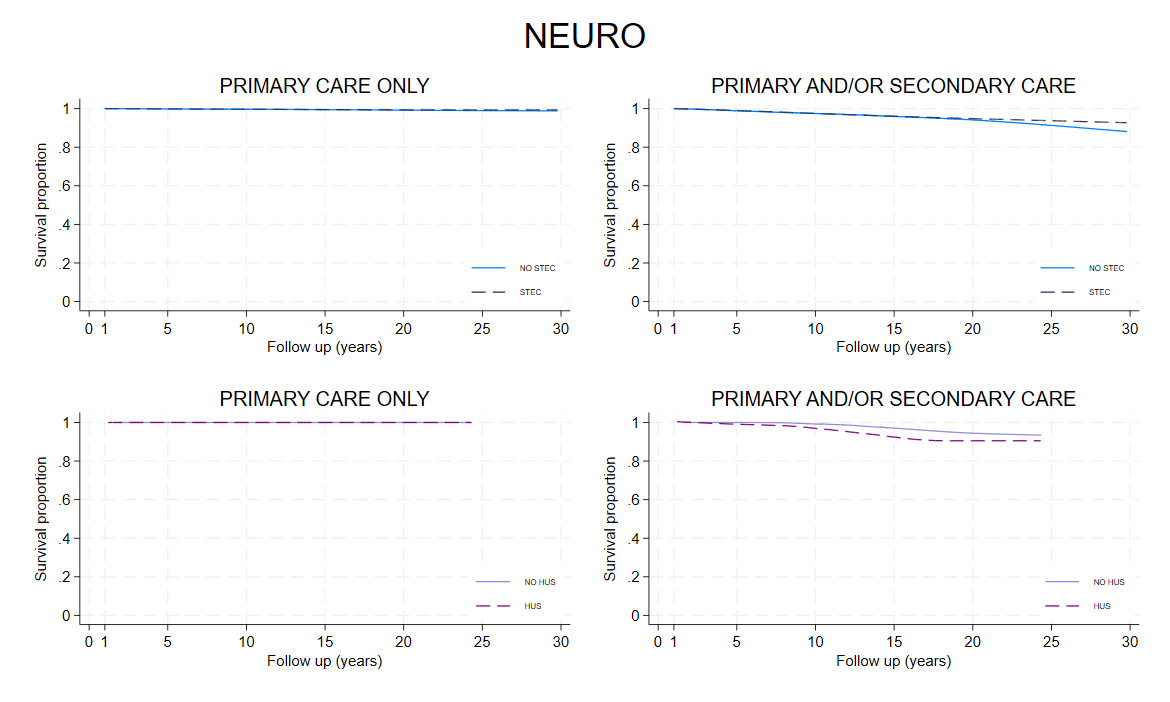


**Appendix G: Smoothed survival curves showing time (years) to first cardiac complication among individuals exposed to Shiga toxin-producing Escherichia coli O157 infection (STEC O157) (first row) or STEC O157 with haemolytic uraemic syndrome (STEC O157-HUS) (second row) at ≥1 year follow-up, January 1990-January 2020, Wales. Censoring events were defined as the first of: death, emigration, or study end (1 January 2020). Outcomes were defined using cardiac clinical code lists for General Practitioner (GP) practice consultations (“Primary care only”) and/or hospital admissions (“Primary and/or secondary care”). Unexposed cohorts were individuals registered with a GP practice in Wales who were the same age and sex as their exposed equivalent at time of STEC O157 positive specimen date or symptom onset**


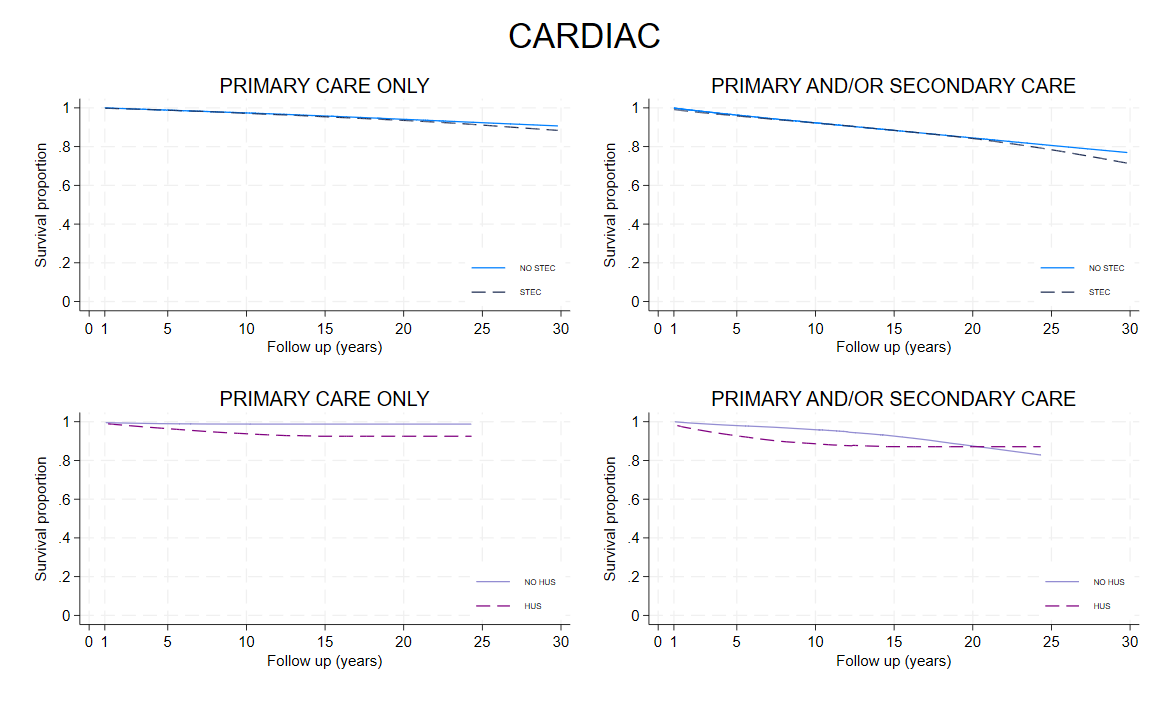


**Appendix H: Smoothed survival curves showing time (years) to first gastrointestinal complication among individuals exposed to Shiga toxin-producing Escherichia coli O157 infection (STEC O157) (first row) or STEC O157 with haemolytic uraemic syndrome (STEC O157-HUS) (second row) at ≥1 year follow-up, January 1990-January 2020, Wales. Censoring events were defined as the first of: death, emigration, or study end (1 January 2020). Outcomes were defined using gastrointestinal clinical code lists for General Practitioner (GP) practice consultations (“Primary care only”) and/or hospital admissions (“Primary and/or secondary care”). Unexposed cohorts were individuals registered with a GP practice in Wales who were the same age and sex as their exposed equivalent at time of STEC O157 positive specimen date or symptom onset**


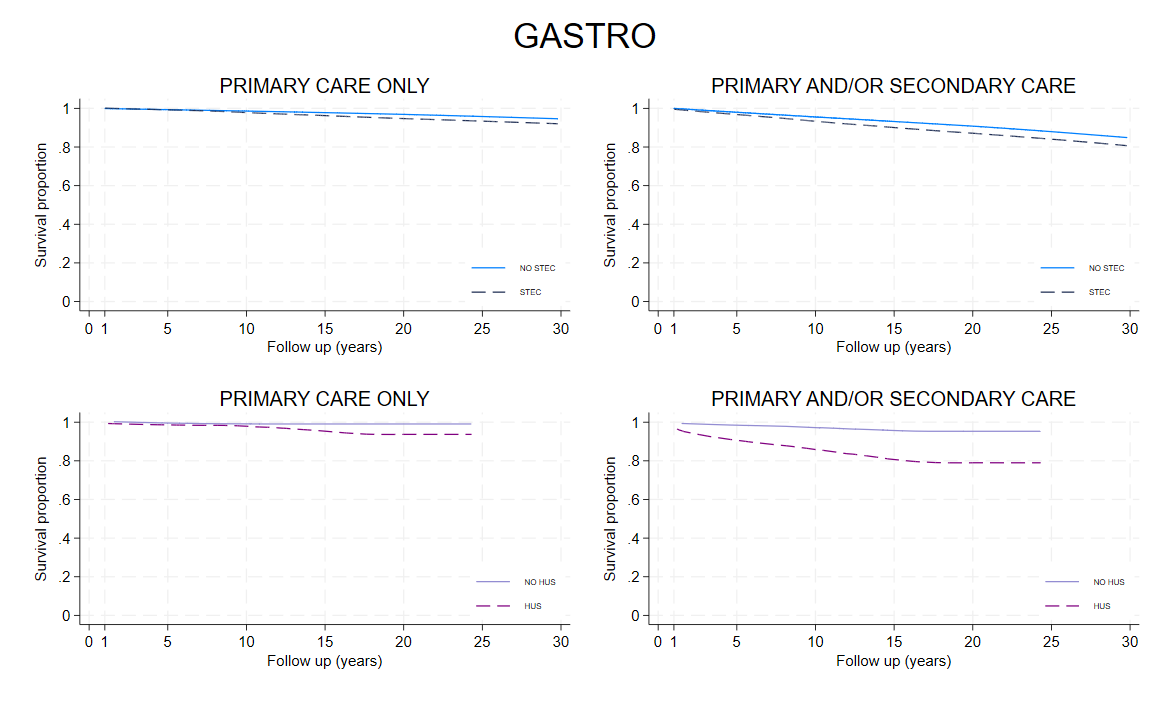


**Appendix I: Smoothed survival curves showing time (years) to first respiratory complication among individuals exposed to Shiga toxin-producing Escherichia coli O157 infection (STEC O157) (first row) or STEC O157 with haemolytic uraemic syndrome (STEC O157-HUS) (second row) at ≥1 year follow-up, January 1990-January 2020, Wales. Censoring events were defined as the first of: death, emigration, or study end (1 January 2020). Outcomes were defined using respiratory clinical code lists for General Practitioner (GP) practice consultations (“Primary care only”) and/or hospital admissions (“Primary and/or secondary care”). Unexposed cohorts were individuals registered with a GP practice in Wales who were the same age and sex as their exposed equivalent at time of STEC O157 positive specimen date or symptom onset**


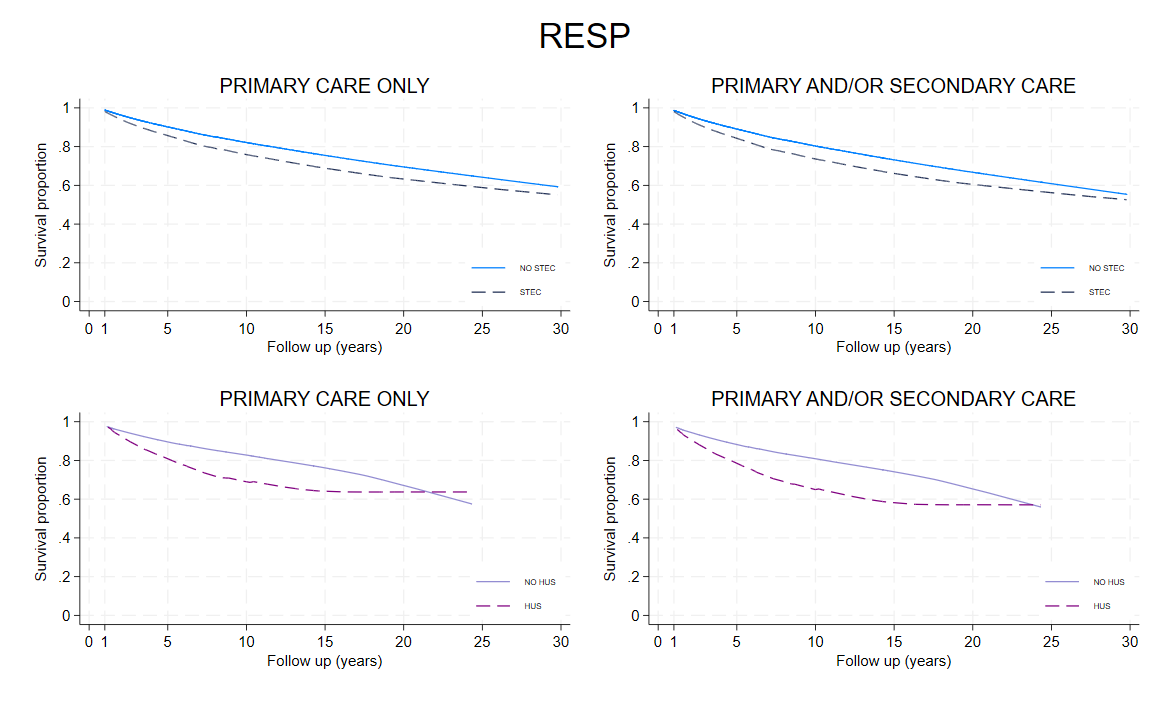


**Appendix J: Smoothed survival curves showing time (years) to first endocrine complication among individuals exposed to Shiga toxin-producing Escherichia coli O157 infection (STEC O157) (first row) or STEC O157 with haemolytic uraemic syndrome (STEC O157-HUS) (second row) at ≥1 year follow-up, January 1990-January 2020, Wales. Censoring events were defined as the first of: death, emigration, or study end (1 January 2020). Outcomes were defined using endocrine clinical code lists for General Practitioner (GP) practice consultations and/or hospital admissions (“Primary and/or secondary care”). Unexposed cohorts were individuals registered with a GP practice in Wales who were the same age and sex as their exposed equivalent at time of STEC O157 positive specimen date or symptom onset**


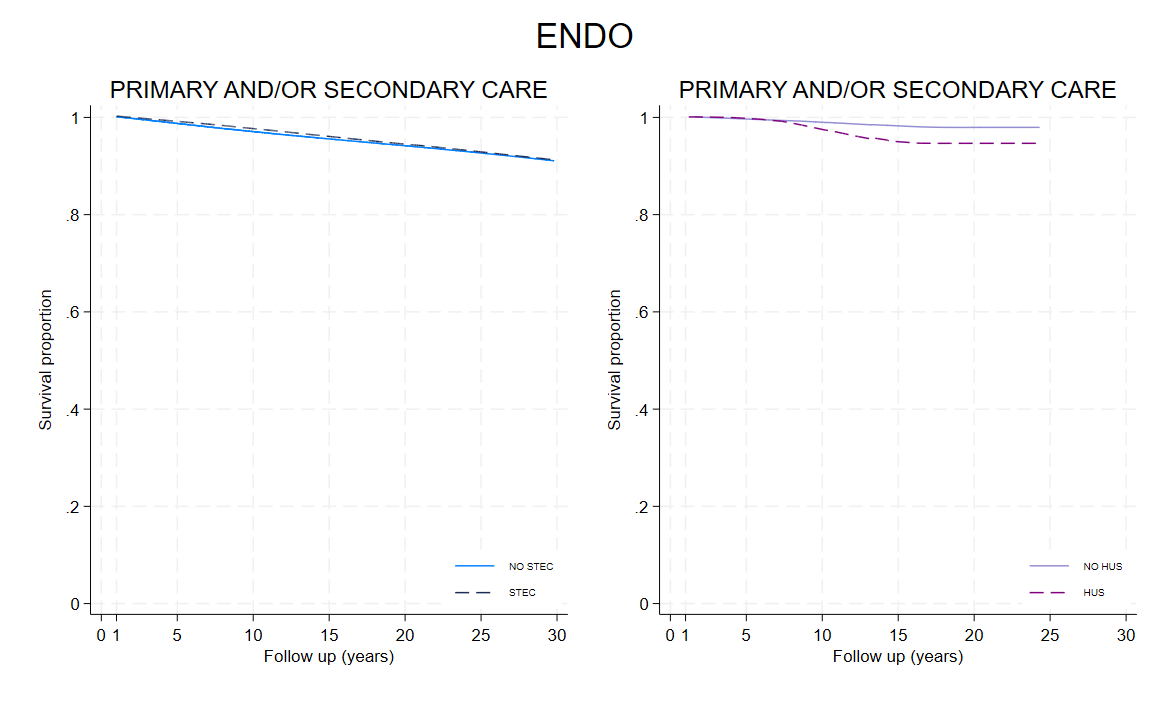


**Appendix K: Crude hazard ratios (HR) and 95% confidence intervals for outcomes (kidney, neurological, cardiac, gastrointestinal, respiratory, endocrine) among individuals exposed to Shiga-toxin producing *Escherichia coli* O157 infection (STEC O157) or STEC O157 with haemolytic uraemic syndrome (STEC O157-HUS), Wales, 1990-2020. Outcomes were defined using clinical code lists for General Practitioner (GP) practice consultations and/or hospital admissions (“Primary and/or secondary care”). Unexposed cohorts were individuals registered with a GP practice in Wales who were the same age and sex as their exposed equivalent at time of STEC O157 positive specimen date or symptom onset**

| **Model** | **Exposure** | **Outcome** | **Exposed** | **Unexposed** | **Hazard ratio** | **Lower CI** | **Upper CI** | **p-value** | **Proportional hazards test p-value** |
| --- | --- | --- | --- | --- | --- | --- | --- | --- | --- |
| **Kidney** |  |  |  |  |  |  |  |  |  |
|  | STEC | Primary care only | 21 | 63 | 1.57 | 0.93 | 2.66 | 0.091 | 0.442 |
|  |  | Primary and/or secondary care | 54 | 185 | 1.20 | 0.87 | 1.67 | 0.268 | 0.945 |
|  | STEC-HUS | Primary care only | <5 | <5 | 3.46 | 0.49 | 24.72 | 0.215 | 0.322 |
|  |  | Primary and/or secondary care | <10 | <10 | 5.51 | 1.58 | 19.18 | 0.007 | 0.186 |
| **Neurological** |  |  |  |  |  |  |  |  |  |
|  | STEC | Primary care only | 5 | 21 | 0.98 | 0.36 | 2.68 | 0.973 | 0.737 |
|  |  | Primary and/or secondary care | 41 | 171 | 0.88 | 0.61 | 1.25 | 0.474 | 0.301 |
|  | STEC-HUS | Primary and/or secondary care | <5 | <5 | 2.30 | 0.51 | 10.36 | 0.279 | 0.275 |
| **Cardiac** |  |  |  |  |  |  |  |  |  |
|  | STEC | Primary care only | 50 | 170 | 1.08 | 0.78 | 1.50 | 0.638 | 0.506 |
|  |  | Primary and/or secondary care | 127 | 460 | 1.08 | 0.87 | 1.34 | 0.466 | 0.585 |
|  | STEC-HUS | Primary care only | <5 | <5 | 5.12 | 1.14 | 22.93 | 0.033 | 0.213 |
|  |  | Primary and/or secondary care | <10 | 13 | 2.54 | 0.93 | 6.93 | 0.068 | 0.108 |
| **Gastrointestinal** |  |  |  |  |  |  |  |  |  |
|  | STEC | Primary care only | 39 | 91 | 1.77 | 1.21 | 2.61 | 0.004 | 0.187 |
|  |  | Primary and/or secondary care | 106 | 278 | 1.59 | 1.26 | 2.01 | 0.000 | 0.679 |
|  | STEC-HUS | Primary care only | <5 | <5 | 7.29 | 0.66 | 80.79 | 0.105 | 0.652 |
|  |  | Primary and/or secondary care | 10 | <10 | 7.71 | 2.63 | 22.59 | 0.000 | 0.952 |
| **Respiratory** |  |  |  |  |  |  |  |  |  |
|  | STEC | Primary care only | 340 | 1016 | 1.37 | 1.20 | 1.56 | 0.000 | 0.032 |
|  |  | Primary and/or secondary care | 371 | 1122 | 1.36 | 1.20 | 1.54 | 0.000 | 0.022 |
|  | STEC-HUS | Primary care only | 20 | 48 | 1.83 | 1.06 | 3.16 | 0.031 | 0.331 |
|  |  | Primary and/or secondary care | <25 | 53 | 1.87 | 1.11 | 3.14 | 0.018 | 0.408 |
| **Endocrine** |  |  |  |  |  |  |  |  |  |
|  | STEC | Primary and/or secondary care | 41 | 177 | 0.98 | 0.69 | 1.40 | 0.924 | 0.070 |
|  | STEC-HUS | Primary and/or secondary care | <5 | <5 | 4.00 | 0.56 | 28.40 | 0.166 | 0.199 |

**Appendix L: Adjusted hazard ratios (HR) and 95% confidence intervals for outcomes (kidney, neurological, cardiac, gastrointestinal, respiratory, endocrine) among individuals exposed to Shiga-toxin producing *Escherichia coli* O157 infection (STEC O157), Wales, 1990-2020. Estimates adjusted for Welsh Index of Multiple Deprivation and co-morbidities at baseline. Outcomes were defined using clinical code lists for General Practitioner (GP) practice consultations and/or hospital admissions (“Primary and/or secondary care”). Unexposed cohorts were individuals registered with a GP practice in Wales who were the same age and sex as their exposed equivalent at time of STEC O157 positive specimen date or symptom onset**

| **Model** | **Outcome** | **Adjusted hazard ratio^1^** | **Lower CI** | **Upper CI** | **p-value** | **Proportional hazards test p-value** |
| --- | --- | --- | --- | --- | --- | --- |
| **Kidney** | Primary care only | 1.89 | 1.07 | 3.34 | 0.028 | 0.970 |
|  | Primary and/or secondary care | 1.19 | 0.85 | 1.66 | 0.303 | 0.855 |
|  |  |  |  |  |  |  |
| **Neurological** | Primary care only | 0.85 | 0.28 | 2.56 | 0.770 | 0.850 |
|  | Primary and/or secondary care | 0.88 | 0.61 | 1.26 | 0.482 | 0.930 |
|  |  |  |  |  |  |  |
| **Cardiac** | Primary care only | 1.04 | 0.75 | 1.44 | 0.825 | 0.136 |
|  | Primary and/or secondary care | 1.09 | 0.88 | 1.36 | 0.419 | 0.275 |
|  |  |  |  |  |  |  |
| **Gastrointestinal** | Primary care only | 1.67 | 1.12 | 2.49 | 0.011 | 0.272 |
|  | Primary and/or secondary care | 1.60 | 1.26 | 2.03 | 0.000 | 0.620 |
|  |  |  |  |  |  |  |
| **Respiratory** | Primary care only | 1.40 | 1.22 | 1.59 | 0.000 | 0.025 |
|  | Primary and/or secondary care | 1.37 | 1.21 | 1.56 | 0.000 | 0.011 |
|  |  |  |  |  |  |  |
| **Endocrine** | Primary and/or secondary care | 1.01 | 0.70 | 1.45 | 0.963 | 0.656 |
| ^1^ Adjusted for deprivation and co-morbidity at baseline | | | | | | |

**REFERENCES**

[19] Mansfield KE, Douglas IJ, Nitsch D, Thomas SL, Smeeth L, Tomlinson LA (2017) Read Code List – ESRD [Data Collection]. <https://datacompass.lshtm.ac.uk/id/eprint/533/>. Accessed 23 September 2024.

[20] Mansfield, K (2023) Clinical codelist - Chronic kidney disease – gold [Online]. <https://datacompass.lshtm.ac.uk/id/eprint/3429/>. Accessed 23 September 2024.

[21] Mansfield KE, Douglas IJ, Nitsch D, Thomas SL, Smeeth L, Tomlinson, LA (2017) Read Code List – Hypertension [Online]. <https://datacompass.lshtm.ac.uk/id/eprint/527/>. Accessed 23 September 2024.

[22] Fonferko-Shadrach B, Lacey A, White C, Powell R, Sawhney I, Lyons R, et al (2017) Validating epilepsy diagnoses in routinely collected data. Seizure 52:195-198. <https://doi.org/10.1016/j.seizure.2017.10.008>

[23] Muzambi R, Warren-Gash C (2020) Clinical codelist - Cognitive impairment incident codes [Online]. <https://datacompass.lshtm.ac.uk/id/eprint/2035/>. Accessed 23 September 2024.

[24] Mansfield KE, Douglas IJ, Nitsch D, Thomas SL, Smeeth L, Tomlinson, LA (2017) Read Code List - Ischaemic Heart Disease [Online]. <https://datacompass.lshtm.ac.uk/id/eprint/529/>. Accessed 23 September 2024.

[25] Malhotra A (2020) Clinical code list - Irritable Bowel Syndrome [Online]. <https://datacompass.lshtm.ac.uk/id/eprint/1923/>. Accessed 23 September 2024.

[26] Iwagami M, Tomlinson LA (2020) Clinical codelist - Inflammatory bowel disease codes [Online]. <https://datacompass.lshtm.ac.uk/id/eprint/1679/>. Accessed 23 September 2024.

[27] Davidson J, Warren-Gash, C (2021) Clinical codelist - CPRD GOLD - acute respiratory infection and influenza/influenza-like-illness codes [Online]. <https://datacompass.lshtm.ac.uk/id/eprint/2192/>. Accessed 23 September 2024.

[31] Stagg V (2006) CHARLSON: Stata module to calculate Charlson index of comorbidity. Statistical Software Components S456719, Boston College Department of Economics, revised 13 September 2017.
